# The role of neutral and adaptive evolutionary processes on patterns of genetic diversity across small cave‐dwelling populations of Icelandic Arctic charr (*Salvelinus alpinus*)

**DOI:** 10.1002/ece3.11363

**Published:** 2024-05-20

**Authors:** Braden J. Judson, Bjarni K. Kristjánsson, Camille A.‐L. Leblanc, Moira M. Ferguson

**Affiliations:** ^1^ Department of Integrative Biology University of Guelph Guelph Ontario Canada; ^2^ Department of Aquaculture and Fish Biology Hólar University Sauðárkrókur Iceland

**Keywords:** colonization history, drift, ecological variation, fish movement, gene flow, geographic distance

## Abstract

Understanding the adaptability of small populations in the face of environmental change is a central problem in evolutionary biology. Solving this problem is challenging because neutral evolutionary processes that operate on historical and contemporary timescales can override the effects of selection in small populations. We assessed the effects of isolation by colonization (IBC), isolation by dispersal limitation (IBDL) as reflected by a pattern of isolation by distance (IBD), and isolation by adaptation (IBA) and the roles of genetic drift and gene flow on patterns of genetic differentiation among 19 cave‐dwelling populations of Icelandic Arctic charr (*Salvelinus alpinus*). We detected evidence of IBC based on the genetic affinity of nearby cave populations and the genetic relationships between the cave populations and the presumed ancestral population in the lake. A pattern of IBD was evident regardless of whether high‐level genetic structuring (IBC) was taken into account. Genetic signatures of bottlenecks and lower genetic diversity in smaller populations indicate the effect of drift. Estimates of gene flow and fish movement suggest that gene flow is limited to nearby populations. In contrast, we found little evidence of IBA as patterns of local ecological and phenotypic variation showed little association with genetic differentiation among populations. Thus, patterns of genetic variation in these small populations likely reflect localized gene flow and genetic drift superimposed onto a larger‐scale structure that is largely a result of colonization history. Our simultaneous assessment of the effects of neutral and adaptive processes in a tractable and replicated system has yielded novel insights into the evolution of small populations on both historical and contemporary timescales and over a smaller spatial scale than is typically studied.

## Graphical abstract

This study evaluates the relative roles of neutral and adaptive evolutionary processes among small populations of Icelandic Arctic charr using a latitudinal series of genetic, phenotypic, and ecological datasets. Over a smaller spatial scale than is typically studied, we demonstrate that contemporary patterns of genetic variation likely reflect localized gene flow and genetic drift superimposed onto a larger scale structure that is largely a result of colonization history.

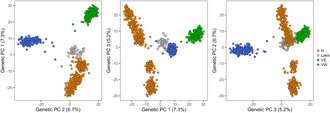

## 1 INTRODUCTION

Understanding the relative roles of the neutral and adaptive processes that drive patterns of genetic diversity in natural populations is a fundamental goal in evolutionary biology. This is particularly important in small populations where adaptability can be limited due to the loss of the genetic variation needed to fuel adaptive responses to environmental change (Hoffmann et al., 2017; Ørsted et al., 2019; Willi & Hoffmann, 2009). Gene flow, however, can counteract the effects of drift by introducing allelic variation but can also constrain local adaptation if the alleles introduced by migrants are maladaptive and/or gene flow is high (Garant et al., 2007). Nevertheless, if selection is sufficiently strong and local ecological conditions favor local alleles over migrant alleles, gene flow will decrease (Nosil et al., 2008; Wang & Bradburd, 2014). Indeed, evidence suggests that small populations can retain adaptive genetic variation (Attard et al., 2022; Ochoa et al., 2020; Yates et al., 2019) arising from factors such as balancing selection (Funk et al., 2016; Schou et al., 2017), relaxed purifying selection (Ferchaud et al., 2020) and varied selection regimes (Wood et al., 2015). Thus, the roles of adaptive processes in the evolution of small populations are highly variable across various contexts.

The processes that interact to partition genetic diversity among populations operate on both historical and contemporary time scales (Fenderson et al., 2020). For instance, colonization history and associated founder and bottleneck events can lead to isolation by colonization (IBC) (Orsini et al., 2013), where populations are clustered genetically cross varying spatial scales (Aldenhoven et al., 2010; Spellman & Klicka, 2007) without associations between patterns of neutral genetic differentiation and ecological variation (Chan & Brown, 2020; Spurgin et al., 2014). For example, many species of northern freshwater fishes have colonized newly accessible habitats after glacial recession and show the genetic footprints of these founding events despite considerable post‐colonization divergence (Bernatchez & Wilson, 1998; Moore et al., 2015; Wilson et al., 2004). However, the effects of more recent colonization events, especially those on smaller spatial scales, on patterns of genetic differentiation are less known (Richardson et al., 2014; Stelkens et al., 2012) even though IBC may be particularly important in freshwater species with limited dispersal options (e.g., Kremer et al., 2017; Shelley et al., 2022).

The evolution of genetic differentiation among populations after colonization is contingent upon contemporary landscape features (Brauer et al., 2018). These can both facilitate or restrict migration and help shape the carrying capacity of the environment, which in turn influences population size and the propensity for drift (Lanier et al., 2015; Salisbury et al., 2016). For instance, species in relatively barrier‐free environments often show limited genetic structuring with high intra‐population genetic diversity, whereas species in more fragmented systems typically have smaller populations with lower genetic diversity (Grummer et al., 2019). When migration is comparatively unrestricted, a process of Isolation by Dispersal Limitation (IBDL) (Orsini et al., 2013) can occur. This is reflected in a pattern of isolation by distance (IBD), wherein genetic differentiation increases as a function of geographic distance (Aguillon et al., 2017; Cameron et al., 2019; Jenkins et al., 2010; Perez et al., 2018). However, the spatial scale at which IBD may be detectable is influenced by IBC, and thus, both need to be considered together.

Understanding the role of selection in shaping patterns of genetic differentiation in small populations is challenging, given the interactive effects of neutral processes. Nevertheless, strong selection can lead to genetic differentiation among populations through the process of Isolation by Adaptation (IBA) (Nosil et al., 2008; Wang & Bradburd, 2014). Accordingly, adaptation leads to reduced gene flow among ecologically divergent habitats because of decreased establishment success of immigrants from different environments (Nosil et al., 2008). A positive association between genetic and ecological variation is potential evidence for IBA (Haileselasie et al., 2016; Shafer & Wolf, 2013; Wang & Bradburd, 2014), but such associations can also arise from autocorrelation with geographic variation (Shafer & Wolf, 2013). Although studies of IBA have typically focused on populations that span large spatial scales and traverse strong ecological gradients (Cooke et al., 2012; Hangartner et al., 2012; Magalhaes et al., 2016), local ecological variation can also help shape genetic population structure (Richardson et al., 2014). This could arise through monopolization, wherein first colonizers rapidly adapt to local conditions and incur a slight selective advantage over subsequent waves of migrants (De Meester et al., 2002, 2016; Orsini et al., 2013). Although potentially important, we know little about the role of IBA in small populations that span subtle ecological gradients (e.g., Attard et al., 2022; Ochoa et al., 2020; Richardson et al., 2014) and the interrelated effects of other processes such IBD and IBC.

The ability to disentangle the effects of IBC, IBDL, and IBA on driving patterns of genotypic differentiation among natural populations is highest in relatively simple and tractable systems and where processes are examined simultaneously (Orsini et al., 2013; Spurgin et al., 2014). Icelandic Arctic charr (*Salvelinus alpinus*) have many features that make them an attractive model system for evolutionary study in this context. The species rapidly colonized Iceland after glacial retreat from a single glacial lineage around 10,000 years ago (Brunner et al., 2001) with subsequent restriction of gene flow and isolation after isostatic rebound (Kapralova et al., 2011; Wilson et al., 2004). More recent geological events such as earthquakes and volcanism (Thorarinsson, 1979), have resulted in colonization of novel habitats (Einarsson et al., 2004). After colonization, Arctic charr in some lakes diversified into discrete resource‐based morphs (e.g., benthic vs pelagic), which are characterized by differences in body shape and size and show reduced gene flow (Brachmann et al., 2021, 2022; Snorasson & Skúlason, 2004). Moreover, considerable variation in body shape and resource use occurs across allopatric populations that is also related to habitat (e.g., ponds vs. streams) (Kristjánsson et al., 2011, 2012). Collectively, these observations suggest that the phenotypic variation observed across the species in Iceland is adaptive in nature but that other factors such as colonization history, drift, and gene flow influenced by the landscape play a role in driving patterns of genetic differentiation among populations.

Our goal is to understand the relative roles of neutral and adaptive evolutionary processes in driving patterns of genetic differentiation in small populations. To address our goal, we are studying a highly replicated system of small populations of cave‐dwelling Arctic charr (*Salvelinus alpinus*) located near Lake Mývatn, Iceland. We previously reported that patterns of microsatellite variation are most likely the result of founder events by lake fish and subsequent drift leading to low intra‐populations genetic diversity coupled with gene flow between nearby populations (Leblanc et al., 2024). In contrast, we found limited evidence of adaptive processes based on the modest amount of variation in body shape explained by ecological factors. Here, we extend our analysis of these populations with a greater number of genetic markers to allow more direct tests of the effects of neutral processes, such as gene flow and genetic drift, over historical and contemporary time scales coupled with a more detailed characterization of local ecological variation and fish movement. This approach provides the opportunity to disentangle the effects of IBC, IBDL, and IBA as well as drift and gene flow on patterns of genetic differentiation in these populations. This simultaneous assessment of the effects of these multiple processes in a tractable and replicated system has the potential to yield novel insights into the evolution of small populations.

## 2 METHODS

### 2.1 Study system

**FIGURE 1:**
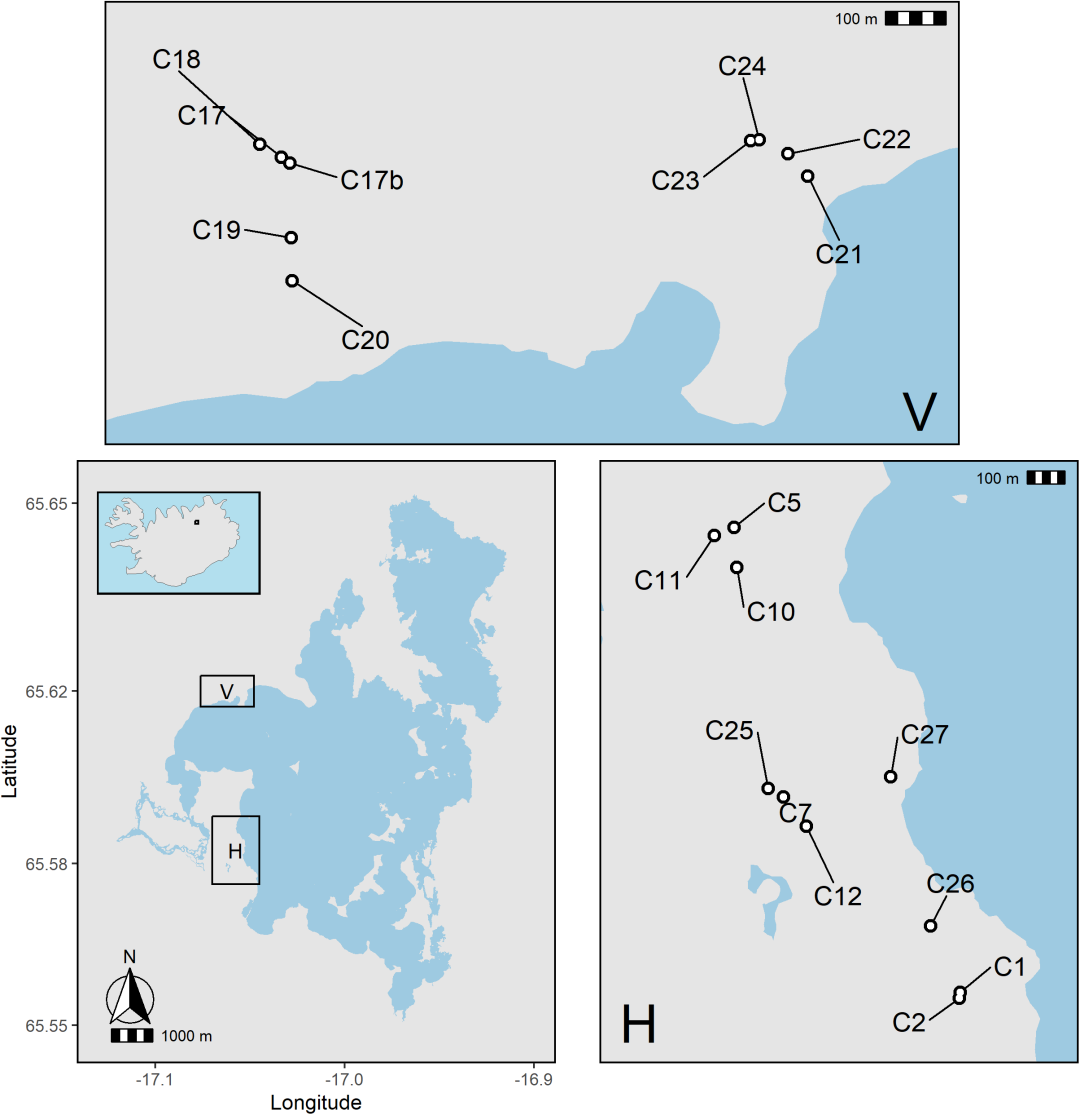
Location of 19 lava caves around Lake Mývatn, Iceland (top left inset) where populations of Arctic charr (*Salvelinus alpinus*) have been monitored since 2012. Nine caves are situated within the Vindbelgur region (V, top) and ten caves are situated within the Haganes region (H, right).

The fish in the current genetic and phenotypic analyses were captured in 2014 and 2019 from 19 lava caves located between 16 and 4279 m apart in the Haganes and Vindbelgur regions near Lake Mývatn, Iceland (Figure 1; Table 1). We refer to the fish living in a cave as a population despite some movement of uniquely tagged fish between some caves (Leblanc et al., 2024). The lava caves were formed approximately 2300 years ago after a reduction in the volume of molten lava under a solidified crust (Thorarinsson, 1979). Fish likely colonized the caves via holes and crevices in the lava after it cooled down, and as these passages filled up with sediments or collapsed due to seismic events, the fish became unable to return to the lake from the caves. Mark‐recapture data between 2012 and 2014 indicate that some fish moved between nearby caves through small subterranean groundwater passages (Leblanc et al., 2024), although it is unknown if fish movement is influenced by season, water flow, or fish size. Moreover, patterns of microsatellite variation in fish captured in 2012 suggest that lake fish colonized the caves and that genetic population sizes, genetic variation, and morphological traits vary among populations.

**TABLE 1:** Geographic information and sample sizes for 1782 Arctic charr (*Salvelinus alpinus*) collected in June and August of 2014 and 2019 from 19 lava caves located in the Haganes (H) and Vindbelgur (V) regions near Lake Mývatn, Iceland.

Population	Region	Geographic cluster	Ecological data?	2014		2019		Total
				June	August	June	August	
Cave 1	H	South (H‐S)	Y	13/13	7/7	7/7	14/14	41/41
Cave 2	H	South (H‐S)	Y	18/18	10/10	13/13	14/14	55/55
Cave 5	H	North (H‐N)	Y	17/25	13/21	8/31	22/22	60/99
Cave 7	H	Central (H‐C)	Y	13/27	17/29	12/22	17/33	59/111
Cave 10	H	North (H‐N)	Y	17/22	13/13	13/18	17/21	60/74
Cave 11	H	North (H‐N)	Y	16/22	15/15	13/13	17/17	60/67
Cave 12	H	Central (H‐C)	Y	21/22	8/8	8/8	12/12	49/50
Cave 17	V	West (V‐W)	N	5/5	9/9	6/6	10/10	30/30
Cave 17b	V	West (V‐W)	Y	7/7	7/7	9/9	16/16	39/39
Cave 18	V	West (V‐W)	Y	17/31	13/19	8/19	22/33	60/102
Cave 19	V	West right (V‐W r)	Y	11/11	2/2	16/16	12/12	41/41
Cave 20	V	West (V‐W)	Y	21/23	9/12	12/22	18/25	60/82
Cave 21	V	East (V‐E)	N	21/22	5/5	10/21	20/26	56/74
Cave 22	V	East (V‐E)	Y	17/17	8/8	11/18	18/37	54/80
Cave 23	V	East (V‐E)	Y	22/27	8/10	10/14	20/28	60/79
Cave 24	V	East (V‐E)	N	17/17	6/6	5/5	13/13	41/41
Cave 25	H	Central (H‐C)	Y	22/134	8/128	20/134	10/87	60/483
Cave 26	H	Central‐South (H‐CS)	N	15/44	15/33	14/31	16/34	60/142
Cave 27	H	Central‐South (H‐CS)	Y	16/20	14/29	17/24	13/19	60/92
Generalist	Lake	Lake Mývatn	N					22/0
Krús	Lake	Lake Mývatn	N					28/0
Total				304/507	188/371	213/424	301/480	1055/1782

*Note*: Ecological data refers to biotic (benthic and aerial) and abiotic variables. The forward slash separates the number of individuals with genotypic and phenotypic data, respectively. Additionally, two morphs (Krús and Generalist, see text for more information) were collected in 2012 from the lake, but these samples have genetic data only.

The fish in the previous (Leblanc et al., 2024) and current study are part of a long‐term project where each population has been sampled nearly exhaustively in both June and August since 2012. The fish were captured by electrofishing and with unbaited fyke and minnow traps. Small numbers of threespine stickleback (*Gasterosteus aculeatus*) occur in some caves, although occurrences are generally rare, and interactions with charr are unknown. Charr were anesthetized in a buffered solution of 2‐phenoxyethanol (300 ppm) and scanned with an electronic tag reader to determine capture history. Untagged fish were PIT‐tagged (HDX; Oregon RFID; 8 mm for fish between 45 and 65 mm fork length (FL) or 12 mm for fish ≥65 mm FL). Fish were weighed (nearest 0.1 g), measured (FL, nearest 1 mm), and photographed on the left lateral side with a scale bar. For newly captured individuals, a small (<30 mg) portion of the upper lobe of the caudal fin was removed. Samples were preserved in 95% ethanol, stored at −20°C, or stored dry at −80°C after ethanol was decanted. For the current study, only a few fish tagged in 2014 (33 or <0.1% of the total number) were recaptured in 2019, suggesting the passage of at least one generation. We excluded these 33 fish from the dataset, leaving 1783 fish for the phenotypic analysis (Table 1). A maximum of 30 individuals from each cave per year had genotypic data available for analysis (Table 2). Additionally, 50 individuals were sampled from Lake Mývatn in June of 2012 (Table 1). Twenty‐eight of these were a benthic‐like morph designated as Krús and were captured using electrofishing along the shore. The remaining fish had a more pelagic morphology (designated as generalists) and were supplied by the Marine and Freshwater Institute, Iceland, after they were captured with gill nets as part of an annual monitoring project (G. Guðbergsson, personal communication). The Krús and generalist fish collectively are hereafter referred to as lake fish in this study.

**TABLE 2:** Estimates of genetic diversity and number of private alleles based on neutral SNPs and census (*N*_c_) and genetic effective (*N*_e_) population sizes of 19 cave (C) populations of Icelandic Arctic charr (*Salvelinus alpinus*).

Population	Region	HLGC	Samples genotyped	Biallelic SNPs	Private alleles	*A*_r_	*H*_o_	*H*_e_	*N*_c_ mean (95% CI)	*N*_e_ mean (95% CI)
C1	H	H	41	937	8	1.470	0.159	0.153	33 (26–49)	13 (11–15)
C2	H	H	55	1030	4	1.500	0.164	0.160	30 (23–49)	18 (16–22)
C5	H	H	60	1054	6	1.543	0.184	0.178	77 (57–116)	43 (34–58)
C7	H	H	59	1213	2	1.621	0.203	0.199	103 (77–147)	48 (36–71)
C10	H	H	60	1056	3	1.547	0.184	0.179	51 (38–83)	22 (19–27)
C11	H	H	60	1093	2	1.566	0.186	0.184	37 (29–55)	17 (15–19)
C12	H	H	49	1149	1	1.591	0.200	0.192	41 (23–81)	11 (10–12)
C17	V	VW	30	874	6	1.448	0.141	0.132	19 (13–34)	10 (9–11)
C17b	V	VW	39	893	3	1.451	0.146	0.140	32 (18–74)	15 (13–18)
C18	V	VW	60	908	11	1.427	0.131	0.127	80 (55–129)	24 (20–31)
C19	V	VW	41	815	2	1.423	0.150	0.141	29 (22–48)	12 (11–14)
C20	V	VW	60	925	3	1.438	0.145	0.143	68 (47–114)	21 (18–25)
C21	V	VE	56	975	0	1.505	0.184	0.174	40 (34–59)	4 (3–4)
C22	V	VE	54	1032	8	1.534	0.183	0.181	65 (40–134)	18 (15–21)
C23	V	VE	60	976	7	1.470	0.157	0.151	53 (38–81)	12 (11–14)
C24	V	VE	41	946	0	1.511	0.178	0.172	39 (21–88)	7 (6–8)
C25	H	H	60	1217	2	1.616	0.199	0.196	365 (298–432)	51 (36–90)
C26	H	H	60	940	0	1.510	0.175	0.171	99 (78–133)	59 (43–97)
C27	H	H	60	1013	5	1.517	0.183	0.174	56 (44–82)	32 (27–39)
Mývatn	L	NA	50	1435	60	1.797	0.239	0.252	NA	NA
Combined										
C1 and C2	H	H	96	1114	15	1.546	0.162	0.158	63 (52–83)	19 (17–21)
C17 and C18	V	VW	90	988	22	1.495	0.134	0.130	96 (71–140)	32 (26–40)

*Note*: Three high‐level genetic clusters (HLGCs) were identified, and population membership to Haganes, Vindbelgur East (VE), and Vindbelgur West (VW) is also indicated (see Section 3 for justification). Genetic diversity was quantified as observed heterozygosity (*H*_o_), expected heterozygosity (*H*_e_), and allelic richness (*A*_r_). Estimates were repeated by grouping individuals from C1 and C2, and C17 and C18 together (termed “combined”) as these populations were not significantly genetically differentiated (see text for more information).

### 2.2 Genetic variation

Genetic variation within and among populations was quantified using single nucleotide polymorphisms (SNPs) (Table 2). Fin samples were rinsed with chilled dH_2_O and digested for 12 h in a proteolytic solution at 37°C. A modified version of a phenol–chloroform DNA extraction protocol (Bardakci & Skibinski, 1994) was performed with phase lock gel tubes (5PRIME, Quantabio). DNA was dissolved in dH_2_O, quantified using an Invitrogen Qubit Fluorometer (ThermoFisher Scientific), and the presence of high molecular weight DNA was visualized via agarose gel electrophoresis before concentrations were standardized to 10 ng/μL. DNA samples were genotyped at the Clinical Genomics Centre at Mount Sinai Hospital (Toronto, Canada) using an Affymetrix Axiom Array for Arctic charr (Nugent et al., 2019). The genotyping array was designed to capture genome‐wide genetic variation and consists of approximately 87k SNPs identified from North American aquaculture strains as well as wild Iceland fish, including small benthic charr from the populations studied here. Additionally, this array has been used to characterize patterns of neutral and adaptive genetic variation in other populations of wild Icelandic fish (e.g., Brachmann et al., 2021).

Using the Axiom Analysis Suite v5.1.1, genotypic output files (*.CEL and *.ARR) were processed following the best practices workflow for a diploid genome, except that the average call rate for a sample to pass was adjusted from 98.5% to 96.5%. Fourteen individuals were discarded due to quality control issues prior to subsequent analyses, leaving 1055 individuals for genetic analyses (Tables 1 and 2). We then implemented conservative SNP filtering steps to minimize the potentially erroneous effects of uninformative loci in inferring population structure (Roesti et al., 2012). Discarded SNPs were either monomorphic, had fewer than three copies of the minor allele (Linck & Battey, 2019), scored inconsistently among twice‐genotyped samples (*N* = 12), or showed non‐Mendelian inheritance in North American families (Danzmann pers. comm.) (Table S1). Biallelic SNPs passing quality control checks were positioned to the *Salvelinus* sp. genome (NCBI GenBank accession: GCF_002910315.2, Christensen et al., 2021) or a pseudochromosome contig hereafter referred to as AC38 (Table S2). This filtering process was undertaken with all fish together for characterization of population structure and then with the cave fish (*N* = 1005) to evaluate the relationships between genetics, ecology, and phenotype (Table S1). After filtering, 1962 and 1752 SNPs remained for the combined and cave‐specific datasets, respectively (Table S1). Genotype missingness was low (<1%), and over 60% of loci were positioned to the genome, although coverage was fewer than one SNP per megabase (Table S2).

### 2.3 Ecological variation

Ecological variation among populations was quantified using four abiotic (Table S3) (water temperature, pH, O_2_ saturation, and conductivity) and eight biotic variables (Table S4) (aerial invertebrates, Chironomidae, Cladocera, Copepoda, Nematoda, Oligochaeta, Ostracoda, Collembola, Hydra, Tardigrada and Coleoptera) that represent potential prey items (B. Kristjánsson, D. Combot, A. Reilent, J. Phillips, and C. Leblanc, unpublished data). Hereafter, the term ecological variables refer to both the abiotic and biotic datasets considered independently. Water temperature was measured four times daily from 2013 with HOBO temperature loggers near the entrance of each cave and approximately 10–20 cm from the bottom. Water pH, conductivity, and oxygen saturation were recorded in June and August from 2013 to 2019 using a calibrated multiparameter probe. Due to the temporal stability of the abiotic parameters (e.g., average 5‐year variance in water temperature ≈0.5°C), we used the mean values over the years for each population. We also calculated the minimum linear distance between each cave and the perimeter of Lake Mývatn.

Benthic and aerial biotic variables were available from 2014 and used as proxies for potential prey item availability (Table S4). For each of the 15 populations (Table 1), three stones were scrubbed to remove the benthic invertebrates, which were then sieved (≤125 μm) and stored in 70% alcohol. The organisms were identified to the lowest taxonomic level possible and enumerated. Similar to Kreiling et al. (2021), the organisms were categorized into seven taxonomic groups plus a group of rare taxa (Table S4) to prevent higher‐level inflation of the data. Arachnid abundances were removed from the dataset, given they were collinear (*r* = .84) with Cladocerans, which are known prey items (Kristjánsson et al., 2012). The input of aerial invertebrates was estimated by placing fall‐in traps built from clear buckets (2300 mL, 196 cm^2^ surface area) at the water surface under a section of the cave entrance. The buckets contained a mixture of 30% propylene glycol, aroma‐free soap, and water. The buckets sample a subset of the aerial invertebrates falling into each cave, given that the cave entrances are always much larger than the size of the bucket. Trap contents were filtered through a 125 μm sieve, and plant materials were removed prior to estimating the total gross volume of invertebrates (ml). Density estimates (ml ·m^−2^) were obtained by dividing invertebrate volumes by the total area of the openings of each cave (B. Kristjánsson, D. Combot, A. Reilent, J. Phillips, and C. Leblanc, unpublished data). Aerial invertebrates were primarily the carcasses of blackflies (Simuliidae) and midges (Chironimidae).

### 2.4 Phenotypic variation

**FIGURE 2:**
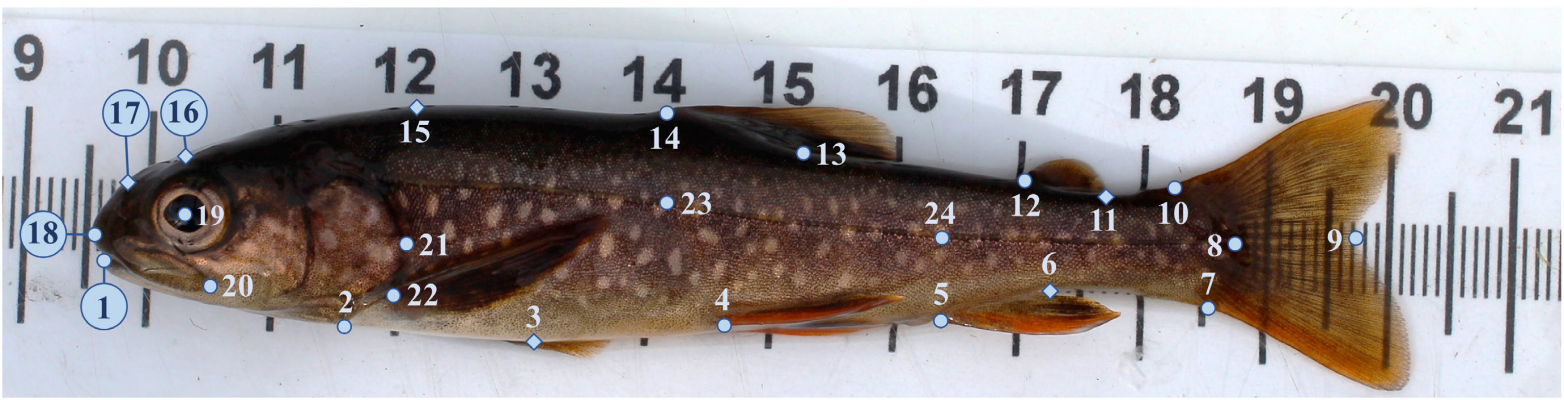
Positions of the landmarks used to characterize shape variation in Arctic charr (*Salvelinus alpinus*), sampled from lava caves around Lake Mývatn, Iceland. Landmarks 6, 11, 15, 16, and 17 are sliding landmarks (diamonds), whereas the remaining 19 landmarks are fixed (circles). Landmarks 23 and 24 were used only for unbending and were removed prior to subsequent analyses. Landmarks 1 to 22 were used to characterize body shape variation, whereas landmarks 1, 2, and 14 through to 22 were used to characterize craniofacial shape variation. Landmark positions are as follows: (1) Anterior tip of the lower mandible, (2) Ventral point of the opercula opening, (3) Half distance between landmarks 2 and 4, (4) Anterior insertion of the pelvic fin, (5) Anterior insertion of the anal fin, (6) Posterior insertion of the anal fin, (7) Ventral insertion of the caudal fin, (8) Posterior point of the hypural bone at the lateral line, (9) Fork of the caudal fin, (10) Dorsal insertion of the caudal fin, (11) Posterior insertion of the adipose fin, (12) Anterior insertion of the adipose fin, (13) Posterior insertion of the dorsal fin, (14) Anterior insertion of the dorsal fin, (15) Posterior edge of the cranium, (16) Top of the cranium at the midpoint of the eye, (17) Middle of the snout, (18) Upper tip of the snout, (19) Center of the bony orbit of the eye, (20) Posterior point of the maxilla, (21) The most posterior point on the curve of the operculum, (22) Anterior insertion of the pectoral fin, (23) Lateral line below the anterior insertion of the dorsal fin, and (24) Lateral line above the anterior insertion of the anal fin.

Body shape variation was characterized by photos of 1783 individuals collected in 2014 and 2019 (Table 1) using 24 homologous landmarks (Figure 2) with the software tpsDIG v2.31 (Rohlf, 2015). Eighteen landmarks were fixed, and six were sliding and positioned along a curve to minimize the average shape difference among specimens. Shape variation associated with dorsoventral arching was minimized using the “unbend” utility in tpsUTIL v1.47 (Rohlf, 2015) with landmarks 23, 24, 8, and 9. Following unbending, landmarks 23 and 24 were removed as these landmarks are not related to body shape variation. All subsequent morphometric analyses were performed using the R package geomorph (Adams & Otárola‐Castillo, 2013). For individuals photographed with their mouth agape, the placement of the landmarks at the anterior tip of the dentary and the posterior end of the maxilla (landmarks 18 and 20, respectively) were imputed on a population‐by‐population basis with the function estimate.missing. We scaled the shape data of all fish to a common size, position, and orientation by performing a generalized Procrustes analysis (GPA). GPA removes the isometric effects (proportions of different body parts remain the same as the fish grows) of body size on shape but retains allometric effects where some parts of the fish grow at different rates than overall body size. Although allometric effects varied among populations, months, and years based on a series of multivariate Procrustes analysis of variance (ANOVAs) (function procD.lm) (Table S5), we retained these effects as they likely represent biologically meaningful developmental variation. Morphometric analyses were performed with whole body shape (22 landmarks) and then craniofacial shape only (11 landmarks; Figure 2) to parse out the effects of short‐term changes in girth due to variation in feeding.

### 2.5 Statistical analyses

All analyses were conducted in R v4.1.1 (R Core Team, 2021) unless indicated otherwise.

### 2.6 Isolation by colonization

We tested for the effects of IBC by characterizing genetic population structure based on neutral loci. This required the identification of loci potentially under selection through outlier loci analysis followed by their removal from the data set (Table S1). We first used pcadapt (Luu et al., 2017) to estimate Mahalanobis distances among SNPs and identify loci associated with population structure (*q*‐value threshold of .05). BayeScan (Foll & Gaggiotti, 2008) was then applied to detect outlier loci with *F*_ST_ values that exceeded background levels of genetic differentiation. BayeScan analyses were performed with 50,000 iterations, 200,000 burn‐in steps, and prior odds of 100 and 1000, representing both relaxed and conservative parameters, respectively. Any locus identified with either approach was removed from the data set. Lastly, to minimize the number of non‐informative SNPs, SNPs in linkage disequilibrium were identified using plink (Purcell et al., 2007). Correlations between pairs of SNPs in a window of 50 loci were estimated, followed by shifts of five loci per iteration, and one SNP from each correlated pair (*R* ^2^ > .80) was discarded at random (approximately 200 SNPs, Table S1).

We tested for significant genetic differentiation among populations and between sampling years within populations with molecular variance (AMOVAs) with the R package poppr (Kamvar et al., 2014). Significance was assessed using ade4 (Dray & Dufour, 2007) following 10,000 repetitions at a significance threshold of *α* = .05. We found no genetic differences between fish sampled in 2014 and 2019 from the same population (Table S6) so all subsequent analyses were based on both years combined. Estimates of expected heterozygosity (*H*_e_), observed heterozygosity (*H*_o_) (R package adegenet (Jombart, 2008), and allelic richness (*A*_r_) (R package hierfstat, Goudet, 2005)) were used to quantify genetic diversity within populations. Genetic differentiation between pairs of populations was tested with Weir and Cockerham's (Weir & Cockerham, 1984) *F*_ST_ through the R package hierfstat (Goudet, 2005) (Table S7). Using the function boot.ppfst and 1000 bootstrap replicates, 95% confidence intervals were calculated. Estimates of F_ST_ were not considered significant if the lower limit was less than or equal to zero.

We identified higher‐level genetic clusters of geographically proximate populations (HLGCs) as potential signatures of IBC (Brunner et al., 2001; Willing et al., 2010) with three analytical approaches. We combined the two groups of lake fish together (*F*_ST_ = 0.058) so that the sample size was comparable to the cave populations; all results were unaffected by the grouping or splitting of these samples. We first identified HLGCs with sparse non‐negative matrix factorization (sNMF) using the R package LEA (Frichot & François, 2015). The sNMF algorithm estimates ancestry coefficients for an unknown number of ancestral gene pools and uses cross‐entropy coefficients to determine the optimal number of genetic clusters. Individual ancestry coefficients were estimated for 1 to 19 ancestral gene pools (*K*) using 50 replicates for each value. Cross‐entropy coefficients were computed for each value of *K*, where lower coefficients indicate better model support. Admixture patterns were visualized with the R package pophelper (Francis, 2017). Individual genotypes were then used to conduct a principal components analysis (PCA), which was followed by the construction of Neighbor‐joining trees. PCAs were performed using the function dudi.pca from the R package ade4 (Dray & Dufour, 2007). Neighbor‐joining trees were constructed with SplitsTree v4.17.1 (Bryant & Moulton, 2004; Huson, 1998) using pairwise Euclidean distances among populations. Using analytical approaches that use both a priori and a posteriori population assignment increases the probability of obtaining a comprehensive picture of genetic population structure and inferred colonization history.

To better understand the effects of IBC on a finer spatial scale, we determined if the observed patterns of interpopulation genetic variation are consistent with the colonization of the caves by lake fish, as reported previously (Leblanc et al., 2024). As before, we expected that the cave populations would share SNPs with the lake fish and have lower levels of genetic diversity, given founder and bottleneck events associated with colonization. If the lava substrate is a physical barrier to colonization by preventing movement, we expected that levels of genetic diversity would be lower in populations more distant from the lake due to lower numbers of founders. We also expected that the magnitude of genetic differentiation (*F*_ST_) between each cave population and the lake would be positively associated with geographic distance, given the assumption that there was a lower probability of fish colonizing the caves further from the lake than the more adjacent ones.

### 2.7 Genetic drift, isolation by distance, and gene flow

We tested for founder effects by searching for signatures of historic bottlenecks. This analysis was first performed across populations within each of the observed HLGCs to detect bottlenecks during the initial phase of colonization and then for each population alone to detect bottlenecks during the later phase of post‐colonization isolation. Using the software Bottleneck v1.2.02 (Cornuet & Luikart, 1996; Piry et al., 1999) and SNPs with a MAF above 5%, heterozygosity expected under the infinite allele model was compared to observed levels with a one‐tailed Wilcoxon signed rank test.

To investigate the effects of genetic drift, we determined if census (*N*_c_) and genetic effective (*N*_e_) population sizes are positively associated with estimates of genetic diversity within populations. Excluding smaller fish (<65 mm FL), *N*_c_ was estimated from individual capture/recapture data for June and August within each of 2014 and 2019 using the Lincoln–Petersen method through the R package FSA (Ogle et al., 2021). The Chapman Modifier was applied to account for small population sizes (i.e., <30) as applicable. The two estimates of *N*_c_ for each population were very similar (adjusted *R* ^2^ = .94, *p* < .001). We used the harmonic mean of the two *N*_c_ estimates to minimize the effects of outlier variation and facilitate comparison with estimates of *N*_e_. *N*_e_ was estimated for each population using temporal changes in allele frequencies between 2014 and 2019 with NeEstimator v.2.1 (Do et al., 2014). SNPs with a minor allele frequency of less than 5% were excluded. Confidence intervals were estimated with Jackknife analyses (Waples & Do, 2008). The associations between *N*_c_, *N*_e_, and all metrics of genetic diversity (*H*_o_, *H*_e_, and *A*_r_) across populations were quantified with simple linear regression models. As C25 has the largest *N*_c_ by far, models with *N*_c_ as a predictor variable were performed with and without this population.

To test for IBD, we determined whether geographic distance is positively associated with genetic distances across pairs of populations using canonical redundancy analyses (RDAs) and spatial eigenvectors (Legendre & Legendre, 2012). Here, the response data was a matrix of principal coordinate analysis (PCoA) values derived from pairwise *F*_ST_ estimates, and the predictor data were a matrix of distance‐based Moran's eigenvector maps (dbMEMs) calculated from pairwise, linear distances between all populations (Borcard & Legendre, 2002). Here, PCoA loadings represent variation in genetic distances between populations. Only PCoA loadings on four axes (each explaining more than 5% of the total genetic variation) were used as response variables, and a forward selection procedure with 10,000 permutations was applied to the model to retain only the most informative dbMEMs (dbMEM3 and 6). To statistically account for the effects of colonization history, the simple RDAs were followed up with partial RDAs wherein a third matrix comprised of ancestry coefficients representing membership probability to each HLGC (*K* = 3, see results) was included. The R package vegan v2.5‐7 (Oksanen et al., 2020) was used to derive dbMEMs and conduct the RDAs and model selection processes.

We investigated the effects of gene flow on population differentiation between pairs of caves by estimating rates in both directions with BA3‐SNPs (Mussmann et al., 2019; Wilson & Rannala, 2003). Using default Mixed chain Monte Carlo parameters (10,000,000 iterations, burn‐in of 1000,000 iterations, and sampling every 100 iterations), the analyses were performed separately for the Haganes and Vindbelgur populations as preliminary runs suggested demographic isolation between regions. BA3‐SNPs‐autotune (Mussmann et al., 2019) was utilized to determine optimal mixing parameters for allele frequencies, inbreeding coefficients, and migration rates for each dataset. Following Wilson and Rannala (2003), each analysis was run five times, and mean values were retained for both migration rates and confidence levels. Estimates with confidence intervals containing negative values were considered not significant. Average rates of contemporary gene flow were compared to migration rates from the mark‐recapture data. Individuals PIT‐tagged in one cave and recaptured in another cave between 2014 and 2019 were considered migrants. A positive association between estimates of gene flow and migration would suggest that migrants are successful breeders.

### 2.8 Isolation by adaptation: Genetic variation

To detect evidence of IBA, we first searched for relationships among patterns of genetic and ecological variation after taking higher‐level genetic structuring (IBC) into account. The analyses were repeated without C25 and by combining pairs of populations with nonsignificant *F*_ST_ values. We first used a distance approach where relationships among pairwise estimates of genetic, ecological, and geographical distances were assessed using multiple matrix regression with randomization (MMRR (Wang, 2013)) as implemented by the R package ecodist v2.0.7 (Goslee & Urban, 2007). These analyses return information on the strength (regression coefficients) and significance (*p*‐values) of each predictor matrix and the fit of the overall model (*R* ^2^). Response variables were pairwise estimates of genetic differentiation (Weir and Cockerham's *F*_ST_), and predictor variables were ecological distance matrices. Distance matrices derived from abiotic and aerial invertebrate data were estimated by using the R function dist to compute pairwise Euclidean distances between populations. Likewise, Hellinger‐transformed benthic invertebrate abundances were used to estimate pairwise Bray‐Curtis (Bray & Curtis, 1957) community dissimilarity indices between populations using the R package vegan. To ensure that variables and regression coefficients are comparable, all distance measures were Z‐transformed before analysis. To aid our interpretation of the results, we tested for autocorrelation among variables by performing a series of ad hoc Mantel tests between all environmental variables and the genetic and geographic distance matrices. This analysis accounts for some of the autocorrelation among the variables analyzed here by simultaneously testing the effects of multiple potentially correlated predictor variables.

We followed up the distance‐based analyses by evaluating relationships among individual genetic, ecological, and geographic variables through a series of canonical RDAs and partial RDAs (pRDAs). The RDA approach has the potential to reveal more subtle associations that may evade detection from distance approaches (Magalhaes et al., 2016). Genetic PCoA scores were used as the response matrix and the predictor matrices were the transformed values from the abiotic, benthic biotic, and aerial biotic variables. To account for colonization history, we performed a series of pRDAs wherein the response data were conditioned upon ancestry coefficients corresponding to each HLGC (*K* = 3, see Section 3). The pRDAs here also account for some autocorrelation among the ecological variables that are correlated with the HLGCs. The performance of each RDA and pRDA was assessed using ANOVA significance testing and model fit estimates (adjusted *R* ^2^).

### 2.9 Isolation by adaptation: Phenotypic variation

We detected large body and craniofacial shape differences between sampling years (Table S8), so we performed the analyses by year or combined depending on the availability of ecological data. Benthic and aerial invertebrate data were only available for 2014, so they were not compared to phenotypic data from 2019. As before, we used both distance‐based and canonical (RDA) approaches to assess relationships. For the distance‐based analyses, response data were morphological distance matrices, and predictor data were the same ecological distance matrices described previously. Pairwise morphological distances were estimated between populations using the function morphol.disparity from the R package geomorph (Adams & Otárola‐Castillo, 2013). This function estimates morphological distances as the average Procrustes variance between groups, and significance is assessed via randomized residual permutations (Zelditch et al., 2004). For the canonical RDAs, the same ecological predictor variables were used as described previously, and the response matrices were comprised of scores along the first three PC axes following a PCA on GPA‐superimposed landmark coordinates. These axes were selected as they each explained approximately 10% of the total phenotypic variation or more and together explained an average of 47% and 62% of the total body and craniofacial shape, respectively. To account for the effects of colonization history, the canonical RDAs were repeated with predictor variables conditioned upon ancestry coefficients representing each of the observed HLGCs (*K* = 3, see Section 3). Model significance was assessed using 10,000 permutations.

## 3 RESULTS

### 3.1 Isolation by colonization

Most of the cave populations differ genetically from each other based on neutral SNP variation. A significant proportion of the total genetic variation (29.8%, *p* < .01) was distributed among populations based on AMOVA (Table S6). In addition, *F*_ST_ values between all but two pairs of populations are highly significant (mean *F*_ST_ = 0.29, standard deviation = 0.11) (Table S7). The most genetically differentiated pair of populations are within the Vindbelgur region (C18 and C23, *F*_ST_ = 0.45). However, pairwise estimates of *F*_ST_ were not significant between two pairs of caves (C1 and C2; C17 and C18) where movement has been detected. All cave populations were highly differentiated from the lake fish (mean *F*_ST_ = 0.33, standard deviation = 0.11, all *p* < .05). Metrics of genetic diversity vary considerably between cave populations, with C18 and C19 having the lowest levels of genetic variation, and the highest estimates of diversity observed in C7 (Table 2). Estimates of genetic diversity were, on average, 15% (*A*_r_) to 45% (*H*_e_) higher among the fish from Lake Mývatn than those in the caves.

**FIGURE 3:**
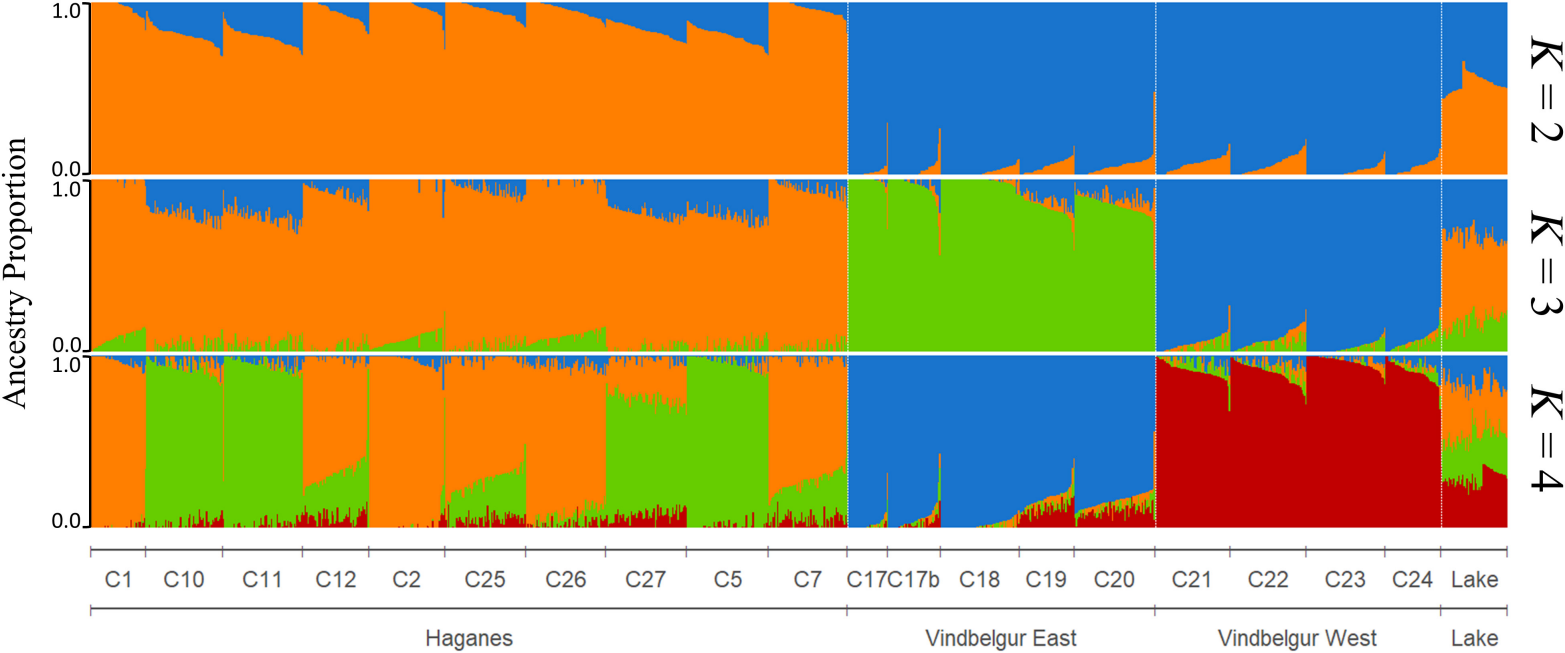
Patterns of genetic admixture among populations of Arctic charr (*Salvelinus alpinus*) sampled from 19 lava caves around Lake Mývatn, Iceland and fish from the lake. Ancestry coefficients were estimated using the sNMF algorithm and neutral SNP frequencies. Populations are distributed across three geographic regions: Haganes, Vindbelgur, and the Lake. Vertical bars represent individuals, and the *y*‐axis depicts the proportion of each genome assigned to each genetic cluster. Patterns of admixture are presented for two, three and four genetic clusters (*K*) (see text for justification).

**FIGURE 4:**
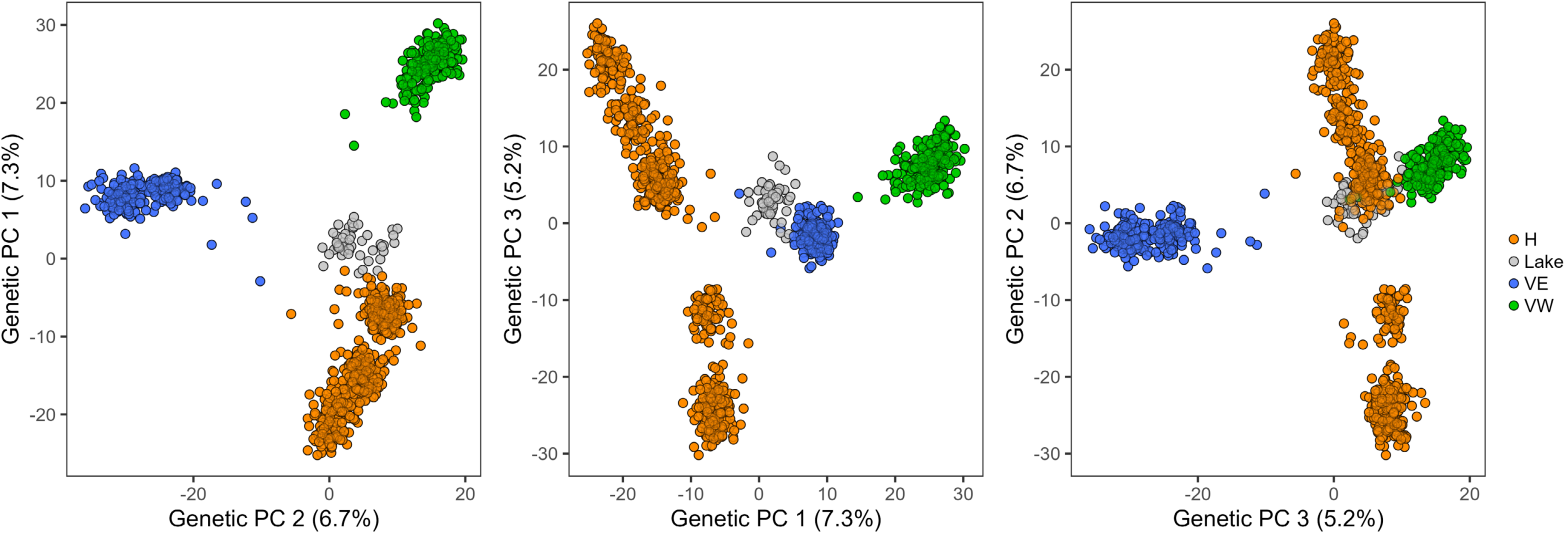
Genetic differentiation among Arctic charr (*Salvelinus alpinus*) sampled from and near Lake Mývatn, Iceland. Principal components analyses were conducted using neutral SNP allele frequencies. Colors indicate high‐level genetic cluster (HLGC; see text for justification) membership to Haganes (H; orange), Vindbelgur East (VE; green), Vindbelgur West (VW; blue), and the lake (gray). Patterns of genetic differentiation are illustrated using PCA scores along the first three axes of variation.

**FIGURE 5:**
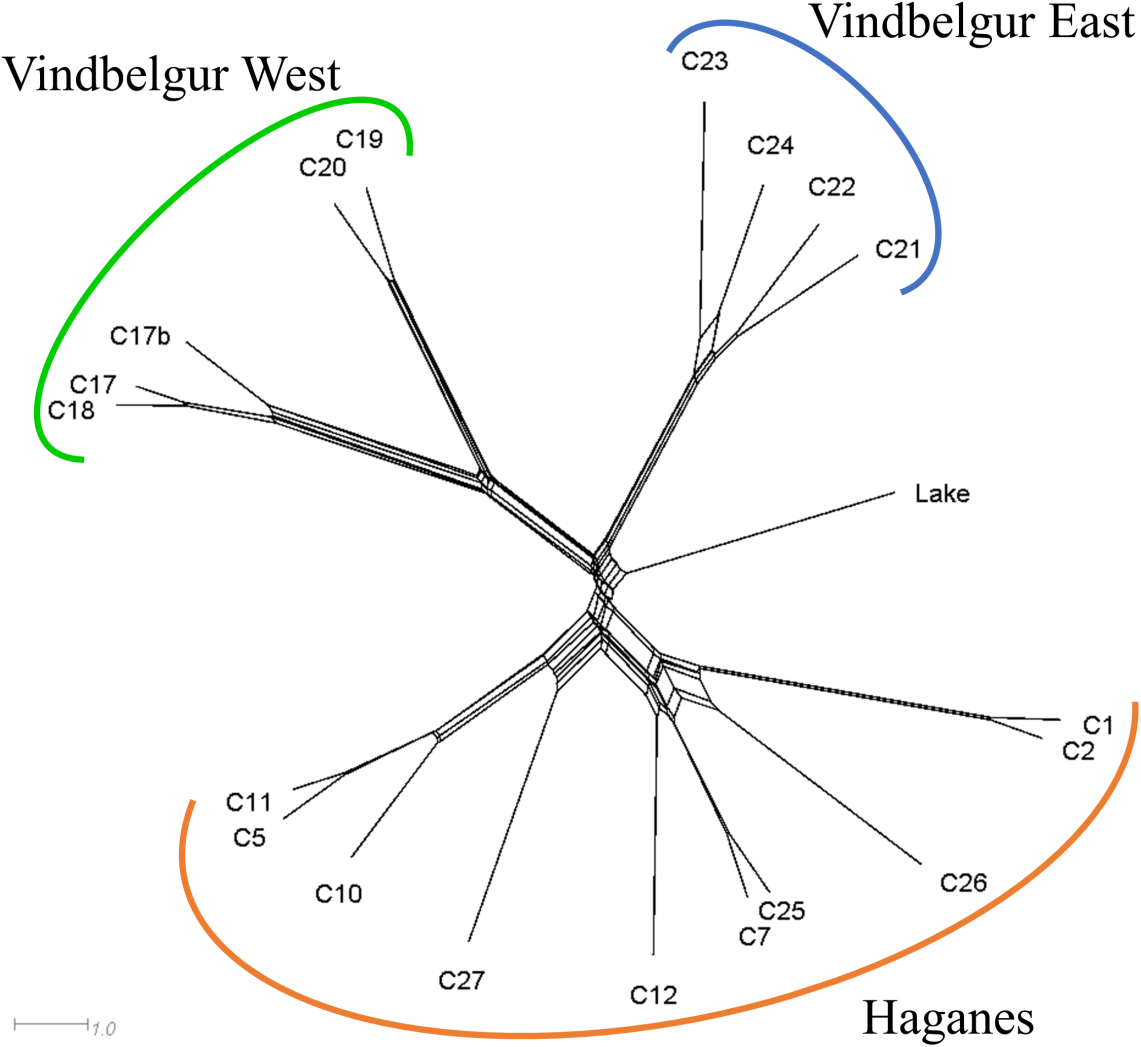
Patterns of genetic differentiation among populations of Arctic charr (*Salvelinus alpinus*), as indicated by a Neighbor‐joining tree. Individuals were sampled from 19 lava caves around Lake Mývatn in addition to samples being collected from within the lake. The tree was constructed from Euclidean distances between all populations. The three high‐level genetic clusters are depicted as Haganes (orange), Vindbelgur West (green), and Vindbelgur East (blue) (see text for justification). The scale bar indicates the branch lengths.

Genetic structuring of populations occurred at both fine and broad geographic scales (Figure 3). Fine‐scale population structure was indicated by the curved distribution of cross‐entropy values estimated in the sNMF analysis, with many large values of *K* performing similarly well (i.e., *K* = 10 through to *K* = 19) (Figure S1). However, the elbow of the distribution suggests higher‐level genetic structuring. For instance, the Haganes and Vindbelgur populations were separated at *K* = 2 with clear differentiation, while *K* = 3 reflected differences between the eastern and western populations in Vindbelgur (Figure 3). In contrast, *K* = 4 indicated a fourth genetic cluster based on relatively small differences between the northern and southern Haganes populations. The existence of three HLGCs is supported by PCA (Figure 4) where the Haganes and Vindbelgur populations were differentiated along the first PC axis (7.3% of the total genetic variation), whereas the second PC axis (6.7%) depicted separation between the eastern and western Vindbelgur populations. Similar to the *K* = 4 sNMF analysis, the third PC axis (5.3%) showed only a modest separation between the northern and southern Haganes populations. The grouping of the Haganes populations into one cluster and the eastern and western populations in Vindbelgur into two additional clusters was also suggested by the Neighbor‐joining tree (Figure 5).

**FIGURE 6:**
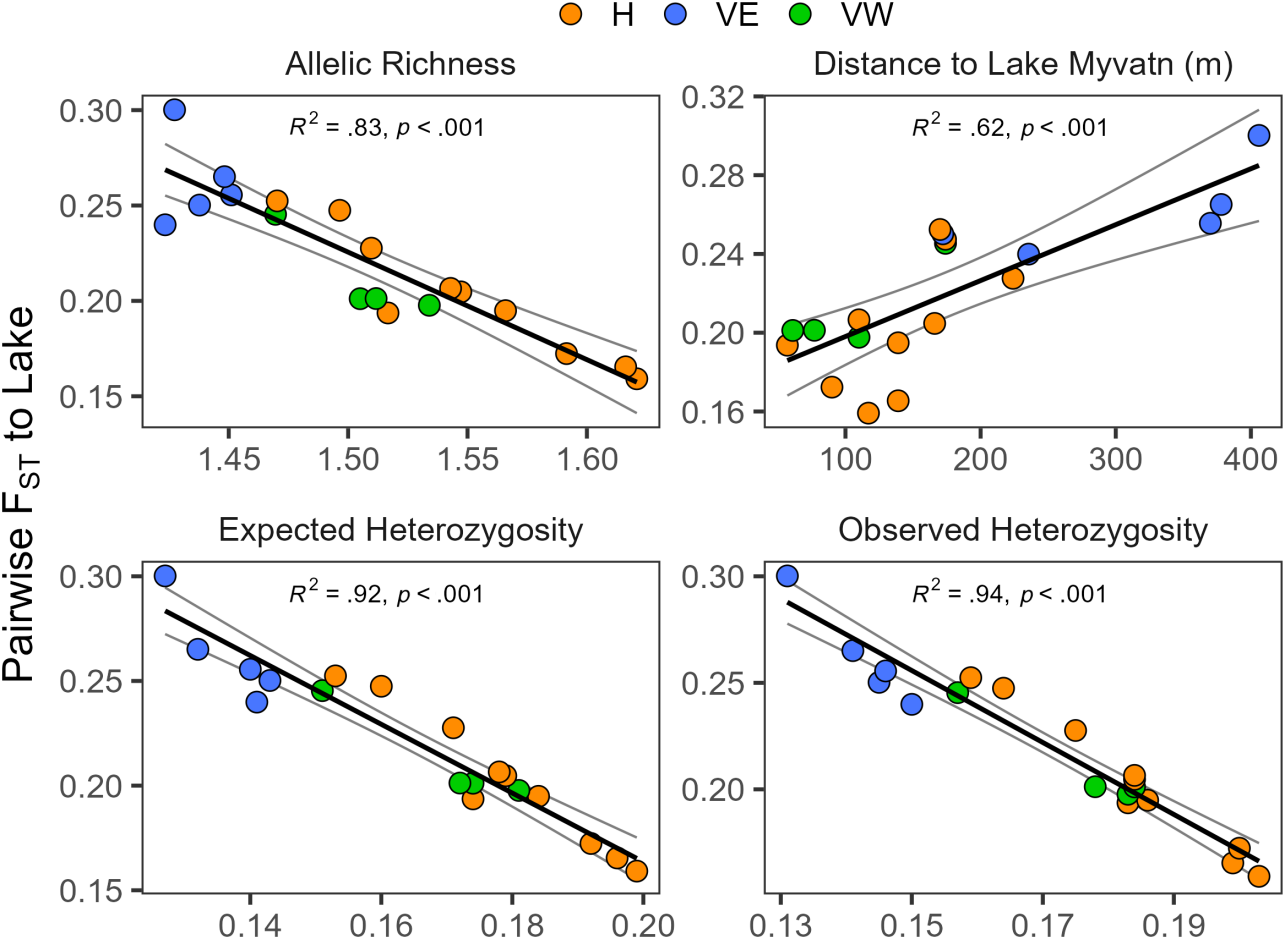
Relationships between estimates of within population genetic diversity and genetic differentiation (Weir and Cockerham's *F*_ST_) for 19 populations of Arctic charr (*Salvelinus alpinus*) from lava caves and charr from Lake Mývatn. Genetic diversity was quantified using expected heterozygosity, observed heterozygosity, and allelic richness. Pairwise estimates of genetic differentiation were also compared to linear distances to the edge of Lake Mývatn. Simple linear regression models were used to assess relationship strength (adjusted *R* ^2^) and significance (*p*‐values). The color of each point reflects membership to each of three high‐level genetic clusters: Haganes (orange), Vindbelgur West (green) and Vindbelgur East (blue) (see text for justification).

Patterns of genetic variation in the cave populations relative to the lake fish support the scenario of colonization of the cave populations by the lake fish suggested previously (Leblanc et al., 2024). First, 3099 of the 3386 alleles (91.5%) found in the cave fish were also detectable in the lake fish, and the lake fish showed greater genetic diversity than the cave populations (Table 2). Second, the degree of differentiation (*F*_ST_) between the cave populations is significantly and positively associated with geographic distance from the lake (*R* ^2^ = .62, *p* < .001) (Figure 6). Finally, there is a significant negative relationship between each metric of within‐population genetic diversity in the cave populations and the degree of differentiation from the lake fish (all *p* < .001).

### 3.2 Genetic drift, isolation by distance, and gene flow

**FIGURE 7:**
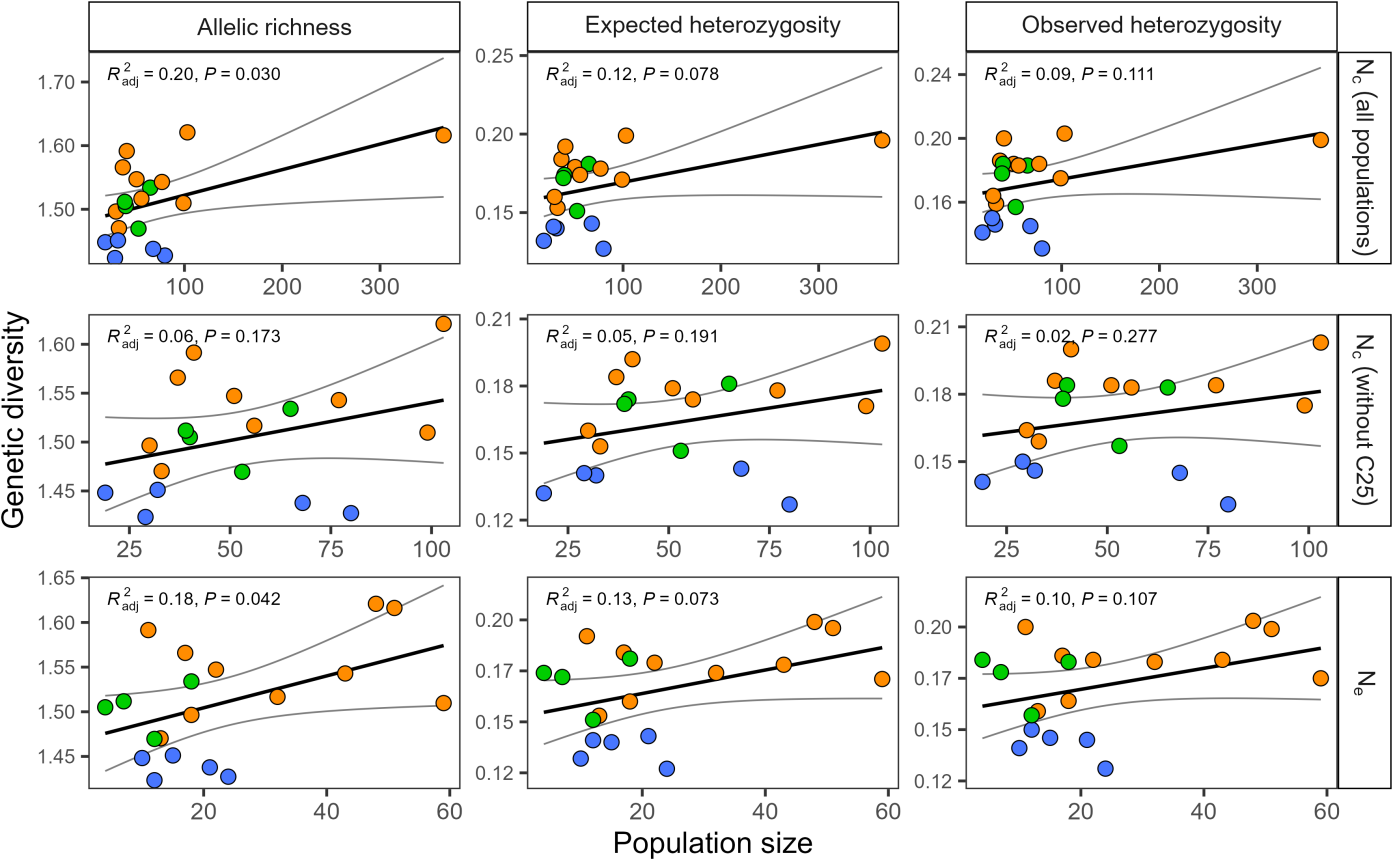
Relationships between census (*N*_c_) and effective (*N*_e_) population sizes and estimates of genetic diversity among 19 populations of Arctic charr (*Salvelinus alpinus*) sampled from lava caves around Lake Mývatn, Iceland. As C25 has a disproportionately large *N*_c_, analyses were repeated with and without this population. Using neutral SNPs, allelic richness (*A*_r_), expected heterozygosity (*H*_e_), and observed heterozygosity (*H*_o_) are used as estimates of genetic diversity. Gray lines indicate 95% confidence intervals. Colors represent membership to the high‐level genetic clusters, identified as Haganes (orange), Vindbelgur East (green), and Vindbelgur West (blue).

We detected signatures of genetic bottlenecks in each of the three HLGCs and all individual populations based on the detection of excess heterozygosity (all *p* < .001). Estimates of *N*_c_ varied markedly among populations, with mean values ranging from 19 to 365 (median = 51) individuals in C17 and C25, respectively (Table 2), where the census size of C25 was much larger than that of any other population. Estimates of *N*_e_ were lower than those of *N*_c_ (average of 3.3‐fold difference), although these estimates are significantly correlated (adjusted *R* ^2^ = .38, *p* = .003). However, there are notable differences in *N*_c_ and *N*_e_ within some caves (Table 2). The relationships between either *N*_e_ or *N*_c_ and estimates of genetic diversity were in the expected positive direction (Figure 7). Both estimates of population size were significant predictors of allelic richness (both *p* < .05) and showed a suggestive relationship with *H*_e_ and *H*_o_ (*p* < .1). However, *N*_c_ was not associated with any estimate of genetic diversity when C25 was removed from the dataset.

We found a pattern of IBD as geographic distance was a significant predictor of genetic differentiation among populations (adjusted *R* ^2^ = .39, *F*_2,16_ = 6.65, *p* < .001) based on RDA and when high‐level genetic structuring (HLGCs) is taken into account (adjusted *R* ^2^ = .45, *F*_2,13_ = 12.06, *p* < .001).

Eleven pairs of neighboring populations (mean geographic distance = 56 m, standard deviation = 29 m) showed statistically significant estimates of contemporary gene flow (Table 3). The estimated rates of effective migration varied from 3% (from C12 to C7) to 25% (from C7 to C25) per generation (Table S9). Estimates of gene flow were asymmetric for many pairs of populations with significant gene flow (Table 3). Finally, estimates of gene flow (average of both directions) are negatively associated with genetic differentiation (*F*_ST_) (adjusted *R* ^2^ = .60, *p* = .003) across the 11 pairs of populations with significant estimates of gene flow.

**TABLE 3:** Number of migrants based on the movement of PIT‐tagged individuals between 2014 and 2019 and population sizes among nine pairs of populations of Arctic charr (*Salvelinus alpinus*) sampled from lava caves around Lake Mývatn, Iceland.

Population pair	Distance (m)	Average *N*_c_	Average *N*_e_	Tagged migrants	Gene flow		*F*_ST_
C1 and C2	16	32	16	15	C1 → C2: 0.161 (0.025)	C2 → C1: 0.198 (0.027)	*NS*
C5 and C10	106	64	32	0	C5 → C10: 0.019 (0.009)	C10 → C5: *NS*	0.061
C5 and C11	57	57	30	11	C5 → C11: 0.176 (0.026)	C11 → C5: 0.159 (0.023)	0.002
C7 and C25	46	234	50	51	C7 → C25: 0.251 (0.030)	C25 → C7: 0.100 (0.018)	0.002
C12 and C7	99	30	11	0	C12 → C7: 0.026 (0.012)	C7 → C12: *NS*	0.096
C17 and C18	42	99	17	14	C17 → C18: 0.231 (0.024)	C18 → C17: 0.011 (0.008)	*NS*
C17 and C17b	17	25	12	4	C17 → C17b: *NS*	C17b → C17: *NS*	0.036
C17b and C18	59	56	19	1	C17b → C18: 0.126 (0.044)	C18 → C17b: 0.014 (0.009)	0.053
C19 and C20	72	44	17	2	C20 → C19: 0.143 (0.025)	C19 → C20: 0.104 (0.023)	0.018
C21 and C22	50	59	11	0	C21 → C22: 0.174 (0.041)	C22 → C21: *NS*	0.040
C22 and C24	54	52	12	0	C22 → C24: 0.067 (0.016)	C24 → C22: 0.045 (0.020)	0.063
C23 and C24	14	46	9	0	C23 → C24: 0.110 (0.040)	C24 → C23: *NS*	0.092

*Note*: Average census and effective population sizes (*N*_c_ and *N*_e_, respectively) are presented for each pair of populations. Average rates of effective migration (percentage per generation (standard deviation)) are presented for both directions. Estimates of gene flow and genetic differentiation (*F*_ST_) are statistically significant (*p* < .05) unless indicated otherwise (*NS*).

The mark‐recapture data indicated that 98 individuals moved between seven pairs of caves at least once between 2014 and 2019 (Table 3). The highest number of migrants was detected between C7 and C25 (*N* = 51), whereas C19 and C20 exchanged only two migrants. Thus, four pairs of populations with significant estimates of contemporary gene flow had no detectable migrants. Significant estimates of gene flow (average in both directions) were positively associated with the number of detected migrants (adjusted *R* ^2^ = .38, df = 9, *p* = .025).

### 3.3 Isolation by adaptation

Abiotic ecological distance was a significant predictor of genetic distance (*p* < .001, Table 4). In contrast, population differences in the benthic and aerial invertebrate ecological distances were not significant predictors of genetic distance. These findings were unaffected by the inclusion or exclusion of C25. Together, the full MMRR distance model explained a considerable proportion of the observed genetic differentiation (adjusted *R* ^2^ = .336). In contrast, the canonical RDAs failed to detect relationships between patterns of genetic differentiation and the abiotic and biotic ecological variables, and this outcome was also unaffected by inclusion or exclusion of data from C25 (Table S10). Accounting for colonization history also failed to detect significant relationships between patterns of genetic differentiation and all ecological variables (Table S10), and these RDAs generally explained a low proportion of the total genetic differences observed (all adjusted *R* ^2^ < .13). The results of both the distance and RDA analyses were unaffected by the grouping or splitting of non‐differentiated population pairs (i.e., C1 and C2, and C17 and C18) (data not shown). We detected significant autocorrelation between abiotic and geographic distances across populations (*r* = .616, *p* < .001) (Table S11).

**TABLE 4:** Relationships between genetic (*F*_ST_), geographic, and ecological distances among 15 populations of Arctic charr (*Salvelinus alpinus*) sampled from lava caves around Lake Mývatn, Iceland.

Model	All populations			Without cave 25		
	*R* ^2^	*β*	*p*(>*F*)	*R* ^2^	*β*	*p*(>*F*)
Genetic differentiation ~						
Abiotic distance	.336	.550	**<.001**	.296	.523	**<.001**
Benthic invertebrates	.336	.035	.682	.296	.039	.670
Aerial invertebrates	.336	−.046	.493	.296	−.022	.796

*Note*: The relationship between the distance matrices was assessed via multiple matrix regression with randomization (MMRR). Response and predictor variables are separated by a tilde (~). Regression coefficients (*β*) and estimates of significance (*p*(>*F*)) are presented for each predictor variable. Genetic distances were represented using Weir and Cockerham's *F*_ST_ and neutral SNPs. Benthic invertebrate dissimilarities were estimated using Bray‐Curtis indices, and Euclidean distances represent distances between abiotic factors and aerial invertebrates. Significant values (*p* < .05) are indicated in bold.

The major axes of body shape variation reflected subtle differences in condition factor, body depth, and head size (Figures S2 and S3). Regardless of the sampling year, the two first PC axes of body shape variation were correlated with body size (using FL as a proxy, all adjusted *R* ^2^ > .15, *p* < .001). The retained craniofacial shape variables also captured fine morphological differences and illustrated differences in snout shape, operculum morphology, and mouth position (Figure S3). The retained shape variables (PCA scores) encompassed more than 45% and 60% of the total body and craniofacial shape variation, respectively (Figures S2 and S3).

The input of aerial invertebrates was associated with variation in body (*R* ^2^ = .031, *β* = .143, *p* = .055) and craniofacial shape (*R* ^2^ = .049, *β* = .0.167, *p* = .033) in 2014, but not 2019 or both years combined based on distance analysis (Table S12). However, the removal of C25 rendered the relationship in 2014 as nonsignificant. This observation suggests that the significance of these relationships is biased by the disproportionate amount of aerial invertebrates that C25 receives during the summer. Estimates of body and craniofacial shape differentiation from 2014 were unrelated to both abiotic and benthic invertebrate differences between populations, regardless of whether C25 was included in the dataset. Similarly, the distance analyses indicate that abiotic differences between populations were unrelated to both body and craniofacial shape differences in 2019 and when data from 2014 and 2019 were grouped together (Table S12).

The canonical RDAs generally corroborated the results of the MMRR analyses (Table S13). Using phenotypic data from 2014, a marginally significant relationship was detected between body shape and the input of aerial invertebrates while accounting for colonization history (adjusted *R* ^2^ = .115, *p* = .060). This relationship, however, was not detectable when C25 was removed from the dataset. Patterns of phenotypic variation from 2019 were not related to patterns of abiotic variation, and this finding was unaffected by the inclusion of C25 or whether colonization history was accounted for. Similarly, abiotic variables were not a significant predictor of phenotypic variation when shape data collected in 2014 and 2019 were grouped together. However, a proportion of variation is explained by differences between the HLGCs with years combined (all *p* < .06, Table S13). This result is detected for both body and craniofacial shape and is unaffected by data from C25. The results produced by both analytical approaches were also unaffected by the grouping or splitting of non‐genetically differentiated population pairs (data not shown).

## 4 DISCUSSION

We assessed the effects of IBC, IBDL, and IBA as well as genetic drift and gene flow on genetic and phenotypic variation in small populations of Icelandic Arctic charr. Large‐scale genetic structuring across populations and their relationships with nearby lake fish suggest that colonization history has contributed to patterns of genetic differentiation. The interaction of genetic drift with gene flow, combined with the physical distance between populations, has influenced patterns of post‐colonization differentiation based on patterns of genetic variation. In contrast, we found little support for IBA as ecological variation is generally unrelated to patterns of genetic and phenotypic variation. Our simultaneous analysis of the effects of multiple processes suggests that patterns of differentiation are largely the product of localized patterns of gene flow and genetic drift arising from small population sizes and physical barriers in the landscape superimposed onto a larger‐scale structure resulting from colonization history. This study emphasizes the role of neutral evolutionary processes operating on historical and contemporary timescales in influencing patterns of genetic and phenotypic variation, which may in turn affect population adaptability.

### 4.1 Isolation by colonization

The detection of three high‐level genetic clusters suggests that the lava caves in the Haganes, Vindbelgur East, and Vindbelgur West geographic regions were colonized in three events. However, the recent and narrow window for colonization of the caves (Thorarinsson, 1979) will make it difficult to determine the relative timing of events with approaches such as demo‐genetic modeling. Success with modeling approaches has been most often achieved with species evolving over longer timescales (Boria et al., 2021; Lanier et al., 2015) and with shorter generation times (Laurent et al., 2011; Rey et al., 2015) than the species studied here. The major issues are that colonization events of a few to hundreds of generations often yield similar genetic signatures (Habel et al., 2014; Launey et al., 2010), and as such, estimates of demographic parameters such as bottleneck timing show relatively large confidence intervals (McCoy et al., 2014; Nunziata et al., 2017). Regardless of the uncertainties in timing, our data indicate that colonization has had a lasting impact on the genetic population of populations across small spatial and temporal scales.

The expanded genetic analysis of the current study supports our earlier suggestion that lake fish were ancestors of the cave populations (Leblanc et al., 2024). First, the cave populations have lower levels of genetic diversity than the lake fish, which is consistent with many island biogeographical studies where the putative source (ancestral) population is more genetically diverse than the smaller descendant populations (Funk et al., 2016; Jordan & Snell, 2008; Wang et al., 2014). Second, cave populations located furthest from the lake are more genetically differentiated from the lake fish and have lower heterozygosity than populations closer to the lake. Third, over 90% of the SNPs found in the cave populations are shared with the lake fish. However, in contrast to the earlier microsatellite study, some of the cave populations contained SNPs that were not detected in the lake fish. Potential explanations for the detection of private alleles are that other (unsampled) source populations contributed to the cave populations and/or the lake fish sample is not representative of either the current population or historical source population. Our findings emphasize the importance of colonization history in understanding contemporary patterns of genetic variation (Caldera & Bolnick, 2008; Machado et al., 2022; Richardson et al., 2014; Spurgin et al., 2014) and are important even at a finer spatial scale than is typically appreciated.

### 4.2 Genetic drift, isolation by distance, and gene flow

Our findings provide collective insight into the interaction of gene flow and genetic drift in shaping patterns of genetic variation in populations after colonization. The observation that evidence of gene flow and detection of fish movement is only detectable between nearby populations suggests that the matrix of lava and sediments in the water in subterranean channels act as a barrier to gene flow. Limited gene flow combined with the small population size estimates, in turn, appears to have affected the propensity for drift to occur. As expected, given that none of the populations had an estimated average *N*_e_ much greater than 50 (Allendorf et al., 2013; Frankham et al., 2014), we found evidence for drift through the detection of signatures of population bottlenecks and the observation that population size is positively associated with intra‐population genetic variation. Our multi‐faceted approach has allowed us to begin to piece together the timing and role of the processes involved in the evolution of these fish, which is not typically possible.

The detection of asymmetric estimates of gene flow between some pairs of populations implies that features of the landscape and associated hydrology are affecting the direction of fish movement. One possible feature is the direction of water flow, which has been used to explain patterns of asymmetric gene flow in fluvial fishes where movement is higher upstream than downstream (Morrissey & De Kerckhove, 2009; Salisbury et al., 2016). Although not a fluvial system, groundwater does flow north from the lake into the Vindbelgur area and west from the lake into the Haganes area (Einarsson et al., 2004; Kristmannsdóttir & Ármannsson, 2004). In fact, gene flow estimates are higher in the direction away from the lake (14.4%), corresponding to the direction of groundwater flow, than toward the lake (6.4%). This difference is significant for nine pairs of populations where significant estimates of gene flow have been detected and where relative orientation to the lake can be inferred (paired *t*‐test, *t* = 2.8, df = 8, *p* = .024). One issue with this explanation is that no water movement/flow has been observed in the cave ponds (personal observation). Water levels are, however, typically higher during the spring thaw, although it is unclear how this influences flow patterns and connectivity between caves. Thus, further characterization of the landscape and hydrology of the Lake Mývatn area will be required to understand the potential drivers of asymmetric gene flow.

The detection of a strong relationship between estimates of genetic and geographic distances between populations provides support for IBDL. Accordingly, reduced gene flow via dispersal limitations may have affected patterns of genetic variation. However, this relationship between genetic and geographic distances can also arise or be reinforced by the effects of sequential founder events along a geographic gradient (Orsini et al., 2013; Pruett & Winker, 2005). In fact, the findings that the populations most distant from the lake are the most differentiated from the lake fish and have the lowest genetic diversity support a model of serial colonization during each of the three suggested colonization events. Similar patterns have been observed in several non‐migratory birds, wherein serial colonization events explain patterns of differentiation between the ancestral source population and the more recently colonized populations (Le Corre & Kremer, 1998; Pruett & Winker, 2005; Sendell‐Price et al., 2021).

### 4.3 Isolation by adaptation

We found limited support for IBA in that neither benthic nor aerial invertebrate availabilities were significant predictors of genetic divergence among populations. However, the distance‐based analyses identified a relationship between abiotic variables and genetic differentiation, unlike the canonical analyses when controlling for presumed colonization. Collectively, this suggests that evidence for IBA in this system is weak at best and is consistent with other studies in wild populations spanning modest ecological gradients (Chan & Brown, 2020; Spurgin et al., 2014). Thus, subtle patterns of ecological variation are less likely to incite adaptive differentiation among populations when the effects of colonization and genetic drift are strong.

The association between genetic and abiotic ecological distances may be the result of autocorrelation with geographic distance. The subdivision of system‐wide genetic variation into clusters of populations, which are in turn located in different geographical regions that vary in abiotic characteristics (Leblanc et al., 2024) could generate autocorrelation. Given that a significant relationship was detected between abiotic and geographic distances, we are therefore unable to disentangle the relative effects of the abiotic ecological variables from geography on genetic variation (Orsini et al., 2013; Shafer & Wolf, 2013). The study of populations where abiotic and geographic distances are not autocorrelated may help parse out the relative effects of geographic distance and abiotic variation in shaping population structure, especially when ecological gradients are subtle.

Features of the available ecological data as well as our genetic and analytical approaches, may also have limited our ability to detect IBA. The ecological differences among the populations may be too small for selection to overcome neutral factors. Furthermore, the ecological variables measured may not have captured evolutionarily relevant ecological factors such as parasites (Hayward et al., 2017; Karvonen & Seehausen, 2012). Likewise, the restriction in the sampling of the biotic variables to a single year will not have characterized variation in annual resource availability (Kreiling et al., 2021; McMeans et al., 2015) such as the input of aerial invertebrates (Einarsson et al., 2004; Ives et al., 2008). Another explanation for the limited evidence for IBA is that the phenotypic variation measured in this study may not be the direct target of selection (Franklin et al., 2018). In fact, body shape variation has been explained by fish community composition and the type of water source in these fish (Kristjánsson & Leblanc, 2018). Finally, the limited genome coverage of genetic markers and the fact that IBA analyses are based on among‐population comparisons could lead to reduced statistical power.

The detection of significant phenotypic differences among populations from the three higher‐level genetic clusters suggests that colonization history may have influenced patterns of contemporary phenotypic variation. This supports a paucity of studies wherein the distribution of complex morphological traits can be attributed to natural founder events (Kolbe et al., 2012; Spurgin et al., 2014) and contrasts the view that founder events and populations bottlenecks are unlikely to leave a detectable imprint on complex phenotypic patterns (Coyne et al., 1997; Zhang, 2018). However, the observed phenotypic differences between fish from genetic clusters may also be a function of the correlation between geographic and abiotic distances, as discussed previously. Thus, understanding the potential role of colonization on phenotypic variation must await additional study that can parse out the effects of potential autocorrelation.

## 5 CONCLUSION

Using comprehensive genetic, phenotypic, ecological, and geographic datasets, our study of small populations of Arctic charr living in lava caves has revealed that contemporary patterns of genetic variation are largely the result of neutral evolutionary processes operating on historical and contemporary time scales despite some variation in ecological conditions. Colonization history appears to have resulted in genetic differentiation among populations in different geographical regions, and the subsequent effects of genetic drift and gene flow influenced by the physical nature of the landscape have likely shaped patterns of post‐colonization divergence. Due to the replicated and tractable nature of the system coupled with long‐term monitoring, we were able to investigate the relative roles of colonization, gene flow, and adaptation at a smaller spatial scale than is typical. Our study shows that historical contingencies may therefore influence contemporary patterns of variation, which may in turn have implications for the long‐term adaptability of small and/or isolated populations. Understanding how the effects of historical events interact with contemporary ecological conditions is an important step in conserving intraspecific biodiversity in fragmented habitats.

## AUTHOR CONTRIBUTIONS

**Braden J. Judson:** Conceptualization (equal); data curation (equal); formal analysis (equal); investigation (equal); methodology (equal); writing – original draft (equal); writing – review and editing (equal). **Bjarni K. Kristjánsson:** Conceptualization (equal); funding acquisition (equal); investigation (equal); methodology (equal); project administration (equal); resources (equal); supervision (equal); writing – review and editing (equal). **Camille A.‐L. Leblanc:** Conceptualization (equal); funding acquisition (equal); investigation (equal); methodology (equal); resources (equal); supervision (equal); writing – review and editing (equal). **Moira M. Ferguson:** Conceptualization (equal); funding acquisition (equal); investigation (equal); project administration (equal); resources (equal); supervision (equal); writing – original draft (equal); writing – review and editing (equal).

## FUNDING INFORMATION

This research was supported by the Icelandic Research Council (RANNIS), Discovery Grants (NSERC, Canada), a Canada Graduate Scholarship (NSERC), and a University of Guelph Graduate Award.

## CONFLICT OF INTEREST STATEMENT

The authors declare no conflicting interests.

## Supporting information


Appendix S1

## Data Availability

Phenotypic and genetic data used in this study have been deposited in the Dryad repository at https://datadryad.org/stash/share/cdphX4ZHruK1S9nWaPBb_OAlxxRaa0PXa6d12_D2z_c.
