# Supplementary material for: The role of neutral and adaptive evolutionary processes on patterns of genetic diversity across small cave‐dwelling populations of Icelandic Arctic charr (Salvelinus alpinus)
Source: Ecol Evol. 2024 May 20;14(5):e11363. doi: 10.1002/ece3.11363 (PMC11103641; doi:10.1002/ece3.11363)
Supplement: Supplementary file 1 — Appendix S1 [file ECE3-14-e11363-s001.docx]

# SUPPORTING INFORMATION: TABLES

Table S1. SNP filtering of genotypic variation in Arctic charr (*Salvelinus alpinus*) sampled from the Mývatn basin, Iceland. Filtering steps were first applied to data from all individuals (Cave and Lake Populations, N = 1055) and cave-dwelling individuals only (Cave Populations Only, N = 1005) (refer to text for more information and sample sizes). Refer to text for information regarding the software and parameters used to filter SNPs.

| **Filtering step** | **Cave and lake populations** | |  | **Cave populations only** | |
| --- | --- | --- | --- | --- | --- |
|  | **Loci removed** | **Loci remaining** |  | **Loci removed** | **Loci remaining** |
| Genotyping array | 0 | 86504 |  | 0 | 86504 |
| Retain recommended loci | 19949 | 66555 |  | 20413 | 66091 |
| Retain biallelic loci | 60701 | 5854 |  | 62659 | 3432 |
| Minor allele count ≥ 3 | 2954 | 2900 |  | 1382 | 2025 |
| Remove non-segregating loci | 65 | 2835 |  | 65 | 1960 |
| Remove inconsistent genotypes | 208 | 1962 |  | 208 | 1752 |
| Remove Pcadapt outliers | 39 | 1945 |  | 28 | 1724 |
| Remove BayeScan outliers | 0 | 1945 |  | 0 | 1724 |
| Subset SNPs in LD | 223 | 1722 |  | 171 | 1553 |

Table S2. Distribution of 1581 biallelic SNPs genotyped in 1005 Arctic charr (*Salvelinus alpinus*) from 19 caves around Lake Mývatn, Iceland. The software BayeScan and pcadapt were employed to identify loci that significantly deviate from neutrality, whereas selectively neutral loci were those not detected by either approach. Where possible, SNPs were positioned to the *Salvelinus* sp. genome (Christensen et al., 2021). SNPs not positioned to the genome were designated to a pseudochromosomal contig, referred here to as AC38.

| **Linkage group** | **Length (Mbp)** | **Outlier SNPs** |  | **Neutral SNPs** | **SNP density (Mb^-1^)** |
| --- | --- | --- | --- | --- | --- |
| AC01 | 58.02 | 0 |  | 19 | 0.33 |
| AC02 | 43.54 | 0 |  | 26 | 0.60 |
| AC03 | 36.00 | 0 |  | 30 | 0.83 |
| AC04p | 28.29 | 0 |  | 22 | 0.78 |
| AC04q.1 | 50.52 | 0 |  | 31 | 0.61 |
| AC04q.2 | 29.60 | 0 |  | 7 | 0.24 |
| AC05 | 37.08 | 1 |  | 38 | 1.02 |
| AC06.1 | 30.25 | 0 |  | 20 | 0.66 |
| AC06.2 | 26.03 | 0 |  | 35 | 1.34 |
| AC07 | 34.30 | 0 |  | 19 | 0.55 |
| AC08 | 54.84 | 0 |  | 49 | 0.89 |
| AC09 | 32.65 | 1 |  | 26 | 0.80 |
| AC10 | 22.46 | 0 |  | 12 | 0.53 |
| AC11 | 51.12 | 2 |  | 52 | 1.02 |
| AC12 | 13.98 | 0 |  | 11 | 0.79 |
| AC13 | 50.98 | 2 |  | 49 | 0.96 |
| AC14 | 54.10 | 0 |  | 32 | 0.59 |
| AC15 | 67.33 | 0 |  | 38 | 0.56 |
| AC16 | 42.87 | 1 |  | 25 | 0.58 |
| AC17 | 41.84 | 0 |  | 22 | 0.52 |
| AC18 | 72.74 | 1 |  | 52 | 0.72 |
| AC19 | 38.23 | 0 |  | 44 | 0.15 |
| AC20 | 80.00 | 4 |  | 69 | 0.86 |
| AC21 | 6.91 | 0 |  | 5 | 0.72 |
| AC22 | 37.60 | 0 |  | 31 | 0.82 |
| AC23 | 49.63 | 2 |  | 24 | 0.48 |
| AC24 | 11.43 | 0 |  | 14 | 1.22 |
| AC25 | 26.20 | 0 |  | 17 | 0.65 |
| AC26 | 49.93 | 0 |  | 41 | 0.82 |
| AC27 | 38.73 | 2 |  | 41 | 1.06 |
| AC28 | 32.73 | 2 |  | 39 | 1.19 |
| AC29 | 40.00 | 0 |  | 28 | 0.70 |
| AC30 | 26.19 | 0 |  | 27 | 1.03 |
| AC31 | 32.01 | 0 |  | 45 | 1.41 |
| AC32 | 38.48 | 0 |  | 42 | 1.09 |
| AC33 | 38.08 | 0 |  | 32 | 0.84 |
| AC34 | 8.96 | 1 |  | 8 | 0.89 |
| AC35 | 21.60 | 0 |  | 6 | 0.28 |
| AC36 | 41.23 | 0 |  | 26 | 0.63 |
| AC37 | 19.55 | 1 |  | 27 | 1.38 |
| AC38 | NA | 6 |  | 565 | NA |

Table S3. Values of abiotic ecological variables for lava caves near Lake Mývatn, Iceland. Water temperature, pH, oxygen saturation and conductivity values are averages from data collected in June and August between 2013 and 2019. Note that water chemistry data are unavailable for Cave 24 and Cave 26.

| **Cave** | **Region** | **HLGC** | **Distance to lake (m)** | **Temperature (^o^C)** | **pH** | **O_2_ saturation (%)** | **Conductivity (µS)** |
| --- | --- | --- | --- | --- | --- | --- | --- |
| C1 | H | H | 170 | 6.7 | 8.6 | 63.3 | 149.2 |
| C2 | H | H | 174 | 5.7 | 8.7 | 57.6 | 149.9 |
| C5 | H | H | 110 | 6.6 | 7.9 | 56.4 | 138.5 |
| C7 | H | H | 117 | 6.4 | 8.6 | 43.1 | 148.3 |
| C10 | H | H | 166 | 6.4 | 8.3 | 65.4 | 154.0 |
| C11 | H | H | 139 | 6.3 | 8.1 | 53.7 | 137.2 |
| C12 | H | H | 90 | 7.0 | 8.3 | 44.4 | 133.2 |
| C17 | V | VW | 378 | 5.9 | 8.6 | 73.3 | 103.8 |
| C17b | V | VW | 370 | 5.8 | 8.6 | 72.9 | 102.9 |
| C18 | V | VW | 406 | 6.1 | 8.5 | 73.7 | 105.5 |
| C19 | V | VW | 236 | 6.2 | 8.6 | 73.4 | 101.7 |
| C20 | V | VW | 172 | 6.7 | 8.4 | 76.8 | 102.4 |
| C21 | V | VE | 61 | 7.6 | 8.5 | 58.2 | 112.0 |
| C22 | V | VE | 110 | 6.6 | 8.4 | 57.6 | 107.8 |
| C23 | V | VE | 174 | 6.9 | 7.7 | 68.5 | 108.3 |
| C24 | V | VE | 162 |  |  |  |  |
| C25 | H | H | 139 | 6.6 | 8.5 | 50.7 | 149.5 |
| C26 | H | H | 102 |  |  |  |  |
| C27 | H | H | 57 | 6.4 | 8.3 | 54.9 | 151.6 |

Table S4. Biotic ecological variables for 15 lava caves around Lake Lake Mývatn, Iceland in the summer of 2014. The input of aerial invertebrates (ml · m^-2^) was estimated using fall-in traps. All other categories are benthic invertebrate densities (individuals per 100 cm^2^) estimated from stone scrubs. For ecological analyses, Collembola, Chaetogaster, Hydra, Tardigrada and Coleoptera were grouped into a single group.

| **Cave** | **Aerial invertebrates** | **Chironimidae** | **Cladocera** | **Copepoda** | **Nematoda** | **Oligochaeta** | **Ostracoda** | **Collembola** | **Chaetogaster** | **Hydra** | **Tardigrada** | **Coleoptera** |
| --- | --- | --- | --- | --- | --- | --- | --- | --- | --- | --- | --- | --- |
| C1 | 27.0 | 12.8 | 0.7 | 99.1 | 14.0 | 5.1 | 19.0 | 0.9 | 0.0 | 0.0 | 1.9 | 0.9 |
| C2 | 412.5 | 3.9 | 11.7 | 31.2 | 0.8 | 4.2 | 5.2 | 0.0 | 0.0 | 0.0 | 0.0 | 0.0 |
| C5 | 120.4 | 2.8 | 19.3 | 37.9 | 2.8 | 2.0 | 43.6 | 0.0 | 0.0 | 0.0 | 1.1 | 0.0 |
| C7 | 4681.3 | 8.9 | 13.4 | 23.1 | 0.0 | 5.6 | 33.7 | 0.0 | 0.0 | 0.0 | 0.0 | 0.5 |
| C10 | 4.2 | 9.4 | 47.3 | 36.0 | 1.8 | 0.0 | 11.2 | 0.2 | 0.0 | 0.0 | 0.0 | 0.0 |
| C11 | 7.2 | 5.4 | 11.0 | 44.0 | 30.8 | 11.2 | 19.6 | 0.0 | 0.2 | 0.0 | 0.3 | 0.1 |
| C12 | 3720.2 | 3.9 | 32.9 | 45.8 | 5.5 | 1.4 | 63.7 | 0.0 | 0.0 | 0.0 | 0.0 | 0.4 |
| C17b | 2960.2 | 6.8 | 4.1 | 71.3 | 17.2 | 23.4 | 87.6 | 0.0 | 0.0 | 0.0 | 0.0 | 0.0 |
| C18 | 300.1 | 6.5 | 4.6 | 32.4 | 7.1 | 5.4 | 49.9 | 0.0 | 0.0 | 0.0 | 0.0 | 0.5 |
| C19 | 1028.7 | 14.1 | 9.0 | 43.1 | 9.5 | 6.1 | 38.9 | 0.0 | 0.0 | 0.0 | 0.0 | 0.0 |
| C20 | 206.3 | 3.2 | 23.8 | 35.2 | 15.1 | 0.2 | 9.2 | 0.0 | 0.0 | 0.2 | 0.0 | 1.9 |
| C22 | 4337.1 | 9.0 | 16.8 | 16.8 | 14.3 | 0.0 | 56.2 | 0.0 | 0.0 | 0.0 | 0.0 | 0.0 |
| C23 | 787.3 | 3.1 | 17.9 | 15.0 | 82.1 | 9.5 | 20.1 | 0.0 | 0.0 | 0.3 | 0.3 | 0.3 |
| C25 | 22192.6 | 3.4 | 68.5 | 68.2 | 30.7 | 6.6 | 73.8 | 0.6 | 0.0 | 0.0 | 0.0 | 0.2 |
| C27 | 2385.5 | 6.9 | 80.2 | 53.9 | 0.0 | 2.6 | 11.2 | 0.0 | 0.0 | 0.0 | 0.3 | 0.0 |

Table S5. Spatial and temporal partitioning of allometric variation of body shape using Procrustes ANOVAs. Shape data were obtained from 1782 Arctic charr (*Salvelinus alpinus*) sampled from 19 lava caves around Lake Mývatn, Iceland. Body shape was characterized using 22 homologous landmarks. Fork length (FL) and centroid size (Csize) are used as proxies for body size. Significance was assessed using 10,000 randomized residual permutations. The degree of significance is indicated by one, two or three asterisks, indicating p values less than 0.05, 0.01 and 0.001, respectively.

| **Model** | **R^2^** | **F** | **Z** | **Pr(>F)** |
| --- | --- | --- | --- | --- |
| Body shape ~ FL | 0.117 | 274.51 | 14.95 | < 0.001 *** |
| Body shape ~ FL * Cave | 0.015 | 1.90 | 6.28 | < 0.001 *** |
| Body shape ~ FL * Year | 0.006 | 13.53 | 7.09 | < 0.001 *** |
| Body shape ~ FL * Month | < 0.001 | 2.45 | 2.26 | 0.0116 * |
| Body shape ~ FL * Year * Cave | 0.014 | 1.89 | 6.49 | < 0.001 *** |
| Body shape ~ FL * Month * Cave | 0.010 | 1.28 | 2.61 | 0.0054 ** |
|  |  |  |  |  |
| Body shape ~ Csize | 0.104 | 242.50 | 14.05 | < 0.001 *** |
| Body shape ~ Csize * Cave | 0.012 | 1.57 | 4.60 | < 0.001 *** |
| Body shape ~ Csize * Year | 0.009 | 21.57 | 8.66 | < 0.001 *** |
| Body shape ~ Csize * Month | 0.001 | 2.51 | 2.54 | 0.0055 ** |
| Body shape ~ Csize * Year * Cave | 0.012 | 1.53 | 4.44 | < 0.001 *** |
| Body shape ~ Csize * Month * Cave | 0.009 | 1.21 | 1.98 | 0.0246 * |

Table S6. Spatial and temporal distribution of genetic variation among 1005 Arctic charr (*Salvelinus alpinus*) sampled from 19 lava caves around Lake Mývatn, Iceland. Patterns of genetic variation were inferred from neutral SNPs using the function poppr.amova from the R package poppr (Kamvar et al., 2014). Significance values were assessed using 10,000 repetitions and a significance threshold of 0.05. Phi (ϕ) is proportional to the degree of differentiation.

| **Group** | **Variance component** | **Variance** | **% of Total** | ***p*-value** | **ϕ** |
| --- | --- | --- | --- | --- | --- |
| Population | Within samples | 306.56 | 71.43 | 0.01 | 0.286 |
|  | Between samples within populations | -5.46 | -1.27 | 0.98 | -0.018 |
|  | Between populations | 128.08 | 29.84 | 0.01 | 0.298 |
| Year | Within samples | 306.56 | 72.57 | 0.01 | 0.274 |
|  | Between samples within years | 115.70 | 27.39 | 0.01 | 0.274 |
|  | Between years | 0.19 | 0.04 | 0.13 | < 0.001 |
| HLGC | Within samples | 306.56 | 65.87 | 0.01 | 0.341 |
|  | Between samples within HLGCs | 53.99 | 11.58 | 0.01 | 0.150 |
|  | Between HLGCs | 104.90 | 22.54 | 0.01 | 0.225 |

Table S7. Genetic differentiation (F_ST_) (Weir and Cockerman 1984) among 19 populations of Arctic charr (*Salvelinus alpinus*) from the Mývatn basin of Northern Iceland. Genetic distances and significance levels were estimated using neutral SNPs. The R package HIERFSTAT (Goudet 2005) was used to estimate F_ST_ and 95% confidence intervals were derived from 1000 bootstrap replicates. Cells shaded gray indicate that differentiation is not significant.


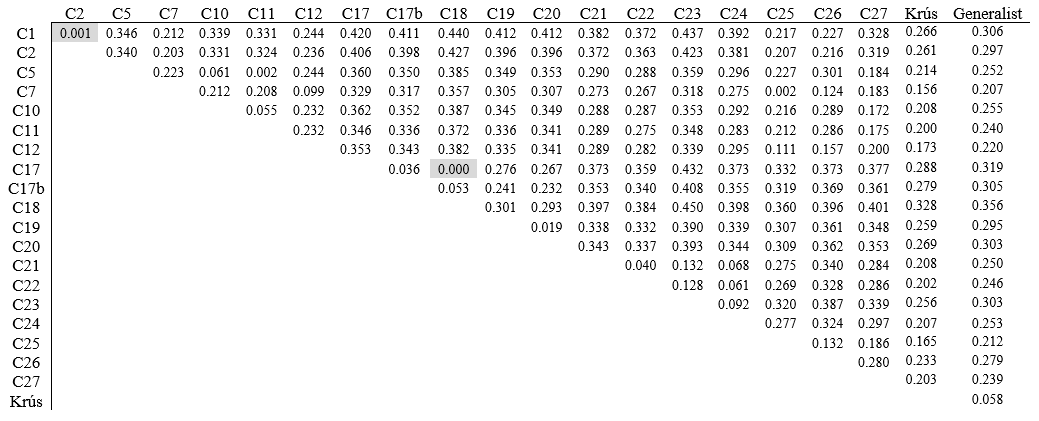


Table S8. Spatial and temporal partitioning of phenotypic variation using a pair of Procrustes ANOVAs. Shape data were obtained from 1782 Arctic charr (*Salvelinus alpinus*) sampled from lava caves around Lake Mývatn, Iceland in June and August of 2014 and 2019. Morphology for body and craniofacial shape are characterized by 22 and 11 landmarks, respectively (Figure 2). The HLGC factor refers to three high-level genetic clusters identified (see Results). Significance was assessed using 10,000 randomized residual permutations and the degree of significance is indicated by one, two or three asterisks, indicating p values less than 0.05, 0.01 and 0.001, respectively.

| **Factor** | **Df** | **R^2^** | **F** | **Z** | **Pr(>F)** |
| --- | --- | --- | --- | --- | --- |
| Body shape |  |  |  |  |  |
| HLGC | 2 | 0.0024 | 2.28 | 2.92 | 0.0012 ** |
| Month | 1 | 0.0032 | 6.01 | 4.64 | < 0.0001 *** |
| Year | 1 | 0.0459 | 86.14 | 11.27 | < 0.0001 *** |
| HLGC * Month | 2 | 0.0013 | 1.23 | 0.83 | 0.2067 |
| HLGC * Year | 2 | 0.0018 | 1.73 | 1.96 | 0.0242 * |
| Month * Year | 1 | 0.0013 | 2.47 | 2.38 | 0.0099 ** |
| HLGC * Month * Year | 2 | 0.0015 | 1.39 | 1.25 | 0.1029 |
|  |  |  |  |  |  |
| Craniofacial shape |  |  |  |  |  |
| HLGC | 2 | 0.0035 | 3.48 | 3.61 | 0.0004 *** |
| Month | 1 | 0.0024 | 4.81 | 3.46 | 0.0003 *** |
| Year | 1 | 0.1006 | 200.72 | 14.88 | < 0.0001 *** |
| HLGC * Month | 2 | 0.0014 | 1.38 | 1.03 | 0.1550 |
| HLGC * Year | 2 | 0.0020 | 1.95 | 1.96 | 0.0242 * |
| Month * Year | 1 | 0.0014 | 2.80 | 2.28 | 0.0115 * |
| HLGC * Month * Year | 2 | 0.0012 | 1.21 | 0.69 | 0.2468 |

Table S9. Effective migration rates (proportion of individuals per generation) between lava cave populations of Arctic charr (*Salvelinus alpinus*) sampled from the Mývatn basin, Iceland. Effective migration rates were estimates using BA3-SNPs (Wilson and Rannala 2003; Mussmann et al., 2019). Estimates (standard deviation) here are average values obtained from 5 runs of each dataset. Significant values are indicated in shaded cells with bold text.


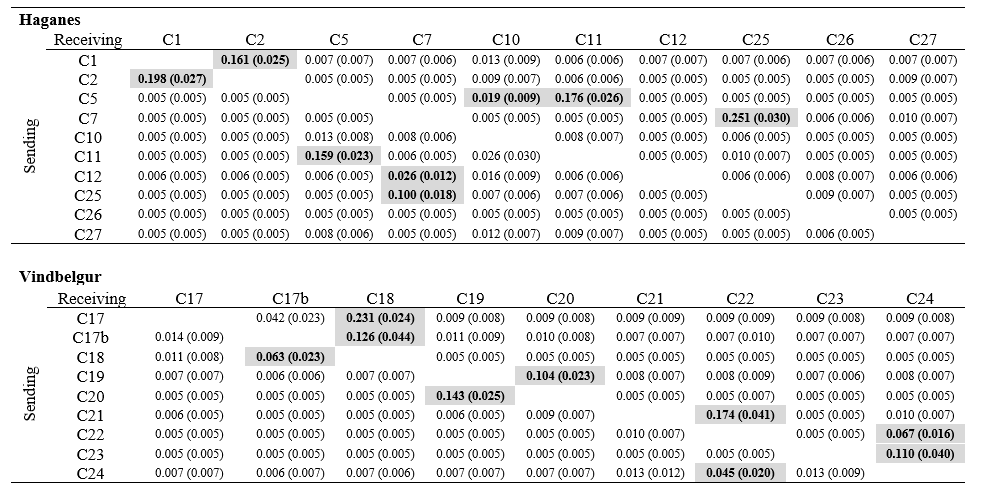


Table S10. Relationships among geographic distance, abiotic and biotic ecological variation, and genetic differentiation (F_ST_) in 15 populations of Arctic charr (*Salvelinus alpinus*) sampled from lava caves around Lake Mývatn, Iceland based on redundancy analyses. All ecological variables were collected in the summer of 2014. Response and each predictor variable are separated by a tilde (~). High-level Genetic Clusters (HLGCs) are comprised of ancestry coefficients reflecting the three main genetic clusters (see text for justification). Analyses were conducted with and without Cave 25 (see text for justification). Partial redundancy analyses (pRDAs) were used to control for the effects of the matrix after the ‘|’ symbol. In this case, the effects of colonization history are accounted for by using ancestry coefficients corresponding to each of three HLGCs. No Significant values (*p* < 0.05) were detected.

| **Model** | **All populations** | | |  | **Without Cave 25** | | |  |
| --- | --- | --- | --- | --- | --- | --- | --- | --- |
|  | **Adjusted R^2^** | **F** | ***P* (>F)** |  | **Adjusted R^2^** | **F** | ***P* (>F)** |  |
| Genetic differentiation |  |  |  |  |  |  |  |  |
| ~ Abiotic factors | 0.129 | 1.517 | 0.139 |  | 0.097 | 1.351 | 0.222 |  |
| ~ Benthic invertebrates | -0.417 | 0.412 | 0.989 |  | -0.490 | 0.389 | 0.993 |  |
| ~ Aerial invertebrates | -0.042 | 0.436 | 0.745 |  | 0.015 | 1.192 | 0.340 |  |
|  |  |  |  |  |  |  |  |  |
| Genetic differentiation |  |  |  |  |  |  |  |  |
| ~ Abiotic factors \| HLGCs | 0.061 | 0.122 | 0.323 |  | 0.087 | 1.513 | 0.198 |  |
| ~ Benthic invertebrates \| HLGCs | -0.594 | 0.312 | 0.987 |  | -0.253 | 0.654 | 0.772 |  |
| ~ Aerial invertebrates \| HLGCs | -0.042 | 0.461 | 0.669 |  | 0.046 | 1.601 | 0.223 |  |

Table S11. Relationships among geographic distance, abiotic and biotic ecological variation among 15 lava caves sampled around Lake Mývatn, Iceland based on simple Mantel tests. Benthic invertebrate dissimilarities were calculated as Bray-Curtis dissimilarity indices. Euclidean distances were computed among aerial invertebrate inputs and abiotic factors. Mantel statistics (r) and *p* values are presented below and above the diagonal, respectively. Significant values (*p* < 0.05) are indicated in bold. Analyses were conducted with and without Cave 25 (see text for justification).

| Dataset | **All populations** | | | |  | **Without Cave 25** | | | |
| --- | --- | --- | --- | --- | --- | --- | --- | --- | --- |
|  | Benthic invertebrates | Aerial invertebrates | Abiotic factors | Geographic distance |  | Benthic invertebrates | Aerial invertebrates | Abiotic factors | Geographic distance |
| Benthic invertebrates |  | 0.757 | **0.036** | 0.081 |  |  | 0.318 | **0.030** | 0.065 |
| Aerial invertebrates | -0.163 |  | 0.726 | 0.758 |  | 0.052 |  | 0.492 | 0.579 |
| Abiotic factors | 0.236 | -0.140 |  | **< 0.001** |  | 0.246 | -0.009 |  | **< 0.001** |
| Geographic distance | 0.138 | -0.080 | 0.616 |  |  | 0.148 | -0.040 | 0.575 |  |

Table S12. Phenotype-environment associations for populations of Arctic charr (*Salvelinus alpinus*) sampled from lava caves around Lake Mývatn, Iceland based on distance analyses. Relationships between phenotypic, ecological, and genetic distance matrices were assessed via multiple matrix regression with randomization (MMRR). Response and predictor variables are separated by a tilde (~). Regression coefficients (β) and estimates of significance (P(>F)) are presented for each predictor variable. The degree of significance is indicated by one or two asterisks, indicating *p*-values less than 0.10 and 0.05, respectively. Benthic invertebrate dissimilarities were estimated using Bray-Curtis indices, and Euclidean distances represent dissimilarities among aerial invertebrates and abiotic variables. Biotic variables were only available from 2014. Genetic distances were calculated using Weir and Cockerham’s F_ST_ and neutral SNPs. Analyses were conducted with and without Cave 25 (see text for justification).

| **Model** | **All populations** | | |  | **Without Cave 25** | | |
| --- | --- | --- | --- | --- | --- | --- | --- |
|  | **R^2^** | **β** | **P(>F)** |  | **R^2^** | **β** | **P(>F)** |
| **2014** |  |  |  |  |  |  |  |
| Body shape ~ |  |  |  |  |  |  |  |
| Abiotic distance | 0.031 | -0.045 | 0.630 |  | 0.025 | -0.105 | 0.290 |
| Benthic invertebrates | “ | 0.024 | 0.812 |  | “ | 0.068 | 0.527 |
| Aerial invertebrates | “ | 0.143 | 0.055 * |  | “ | 0.093 | 0.286 |
|  |  |  |  |  |  |  |  |
| Craniofacial shape ~ |  |  |  |  |  |  |  |
| Abiotic distance | 0.049 | 0.071 | 0.440 |  | 0.007 | 0.065 | 0.529 |
| Benthic invertebrates | “ | -0.042 | 0.677 |  | “ | 0.023 | 0.832 |
| Aerial invertebrates | “ | 0.167 | 0.033 ****** |  | “ | -0.039 | 0.670 |
| **2019** |  |  |  |  |  |  |  |
| Body shape ~ |  |  |  |  |  |  |  |
| Abiotic distance | 0.011 | -0.099 | 0.291 |  | 0.011 | -0.098 | 0.323 |
|  |  |  |  |  |  |  |  |
| Craniofacial shape ~ |  |  |  |  |  |  |  |
| Abiotic distance | 0.015 | 0.117 | 0.192 |  | 0.016 | 0.125 | 0.202 |
|  |  |  |  |  |  |  |  |
| **2014 and 2019 combined** |  |  |  |  |  |  |  |
| Body shape ~ |  |  |  |  |  |  |  |
| Abiotic distance | 0.013 | -0.098 | 0.241 |  | 0.014 | -0.105 | 0.236 |
|  |  |  |  |  |  |  |  |
| Craniofacial shape ~ |  |  |  |  |  |  |  |
| Abiotic distance | 0.075 | 0.078 | 0.364 |  | 0.002 | 0.042 | 0.652 |

Table S13. Phenotype-environment associations for populations of Arctic charr (*Salvelinus alpinus*) sampled from lava caves around Lake Mývatn, Iceland based on redundancy analyses (RDAs). Relationships were detected between multivariate phenotypic data (first three PC axes) and groups of ecological variables. Response and each predictor variable are separated by a tilde (~). The degree of significance is indicated by one or two asterisks, indicating *p*-values less than 0.10 and 0.05, respectively. High-level Genetic Clusters (HLGCs) are comprised of ancestry coefficients reflecting membership to the three main genetic clusters (see text for justification). Partial redundancy analyses were used to control for the effects of the matrix after the ‘|’ symbol. Models were assessed with phenotypic data from both sampling years together and then separate by year. Analyses were conducted with and without Cave 25 (see text for justification).

| **Model** | **All populations** | | | |  | **Cave 25 excluded** | | |
| --- | --- | --- | --- | --- | --- | --- | --- | --- |
|  | **Adjusted R^2^** | | **F** | ***p* value** |  | **Adjusted R^2^** | **F** | ***p* value** |
| **2014** | |  |  |  |  |  |  |  |
| Whole body shape | |  |  |  |  |  |  |  |
| ~ Abiotic factors | | 0.040 | 1.144 | 0.348 |  | -0.026 | 0.917 | 0.522 |
| ~ Benthic invertebrates | | -0.086 | 0.842 | 0.643 |  | -0.136 | 0.778 | 0.735 |
| ~ Aerial invertebrates | | 0.010 | 1.136 | 0.374 |  | 0.040 | 1.535 | 0.188 |
| ~ HLGCs | | 0.093 | 1.715 | 0.153 |  | 0.010 | 1.720 | 0.159 |
|  | |  |  |  |  |  |  |  |
| Whole body shape | |  |  |  |  |  |  |  |
| ~ Abiotic factors \| HLGCs | | -0.015 | 0.949 | 0.534 |  | -0.089 | 0.75 | 0.648 |
| ~ Benthic invertebrates \| HLGCs | | 0.104 | 1.223 | 0.359 |  | 0.193 | 1.429 | 0.290 |
| ~ Aerial invertebrates \| HLGCs | | 0.115 | 2.742 | 0.060 * |  | -0.080 | 0.102 | 0.948 |
|  | |  |  |  |  |  |  |  |
| Craniofacial shape | |  |  |  |  |  |  |  |
| ~ Abiotic factors | | -0.045 | 0.850 | 0.553 |  | -0.054 | 0.832 | 0.564 |
| ~ Benthic invertebrates | | 0.248 | 1.661 | 0.214 |  | 0.202 | 1.468 | 0.314 |
| ~ Aerial invertebrates | | 0.022 | 1.313 | 0.244 |  | 0.022 | 1.290 | 0.254 |
| ~ HLGCs | | 0.089 | 1.683 | 0.189 |  | 0.100 | 1.772 | 0.171 |
|  | |  |  |  |  |  |  |  |
| Craniofacial shape | |  |  |  |  |  |  |  |
| ~ Abiotic factors \| HLGCs | | -0.114 | 0.69 | 0.681 |  | -0.025 | 0.920 | 0.515 |
| ~ Benthic invertebrates \| HLGCs | | 0.262 | 1.646 | 0.250 |  | 0.307 | 1.870 | 0.156 |
| ~ Aerial invertebrates \| HLGCs | | 0.013 | 1.164 | 0.308 |  | 0.072 | 2.036 | 0.160 |
|  | |  |  |  |  |  |  |  |
| **2019** | |  |  |  |  |  |  |  |
| Whole body shape | |  |  |  |  |  |  |  |
| ~ Abiotic factors | | -0.050 | 0.834 | 0.612 |  | 0.023 | 1.075 | 0.424 |
| ~ HLGCs | | -0.056 | 0.628 | 0.690 |  | -0.020 | 0.870 | 0.542 |
| ~ Abiotic factors \| HLGCs | | 0.011 | 1.031 | 0.455 |  | 0.110 | 1.333 | 0.283 |
|  | |  |  |  |  |  |  |  |
| Craniofacial shape | |  |  |  |  |  |  |  |
| ~ Abiotic factors | | -0.083 | 0.733 | 0.685 |  | -0.022 | 0.929 | 0.521 |
| ~ HLGCs | | -0.110 | 0.320 | 0.899 |  | -0.084 | 0.499 | 0.829 |
| ~ Abiotic factors \| HLGCs | | 0.215 | 1.681 | 0.173 |  | 0.212 | 1.709 | 0.139 |
|  | |  |  |  |  |  |  |  |
| **2014 and 2019 combined** | |  |  |  |  |  |  |  |
| Whole body shape | |  |  |  |  |  |  |  |
| ~ Abiotic factors | | 0.172 | 1.725 | 0.146 |  | 0.200 | 1.813 | 0.162 |
| ~ HLGCs | | 0.194 | 2.682 | 0.052 * |  | 0.244 | 3.10 | 0.052 * |
| ~ Abiotic factors \| HLGCs | | 0.052 | 1.206 | 0.378 |  | 0.081 | 1.330 | 0.312 |
|  | |  |  |  |  |  |  |  |
| Craniofacial shape | |  |  |  |  |  |  |  |
| ~ Abiotic factors | | 0.097 | 1.377 | 0.262 |  | 0.088 | 1.313 | 0.310 |
| ~ HLGCs | | 0.225 | 3.037 | 0.054 * |  | 0.236 | 3.011 | 0.059 * |
| ~ Abiotic factors \| HLGCs | | -0.031 | 0.883 | 0.537 |  | -0.093 | 0.701 | 0.685 |
|  | |  |  |  |  |  |  |  |

# SUPPORTING INFORMATION: FIGURES


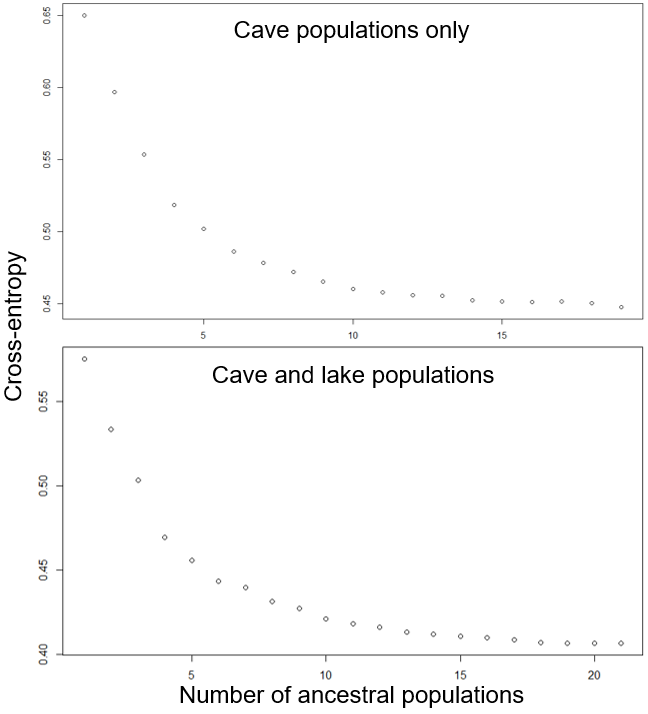


Figure S1. Detection of high-level genetic clusters among Arctic charr (*Salvelinus alpinus*) sampled the Mývatn region of northeastern Iceland. Cross-entropy values were estimated from samples from 19 lava caves around Lake Mývatn (top). This analysis was conducted a second time using data from all cave-dwelling individuals and samples from within the lake (bottom). As indicated by the elbow in the cross-entropy values, there are high-level genetic clusters present at K = 3 (see text for justification).


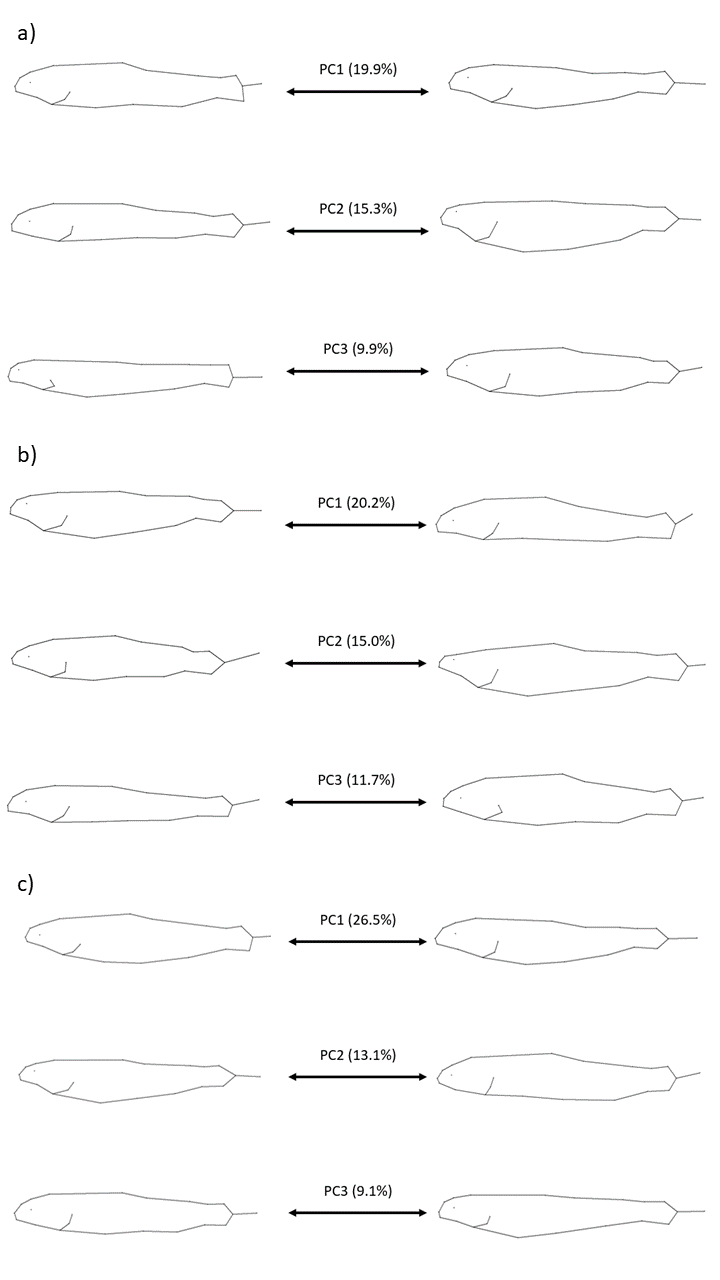


Figure S2. Body shape variation among Arctic charr (*Salvelinus alpinus*) sampled from lava caves around Lake Mývatn in 2014 and 2019. Data are presented separately for both sampling years grouped together (a), as well as 2014 and 2019 separately (b and c, respectively). Variation in body shape was assessed using 22 homologous landmarks. Superimposed landmark coordinates were subjected to a principal components analysis, where first three axes were retained for each grouping. Smaller values are on the left, whereas larger values are on the right of each axis. The proportion of the total phenotypic variation explained by each axis is depicted above each arrow.


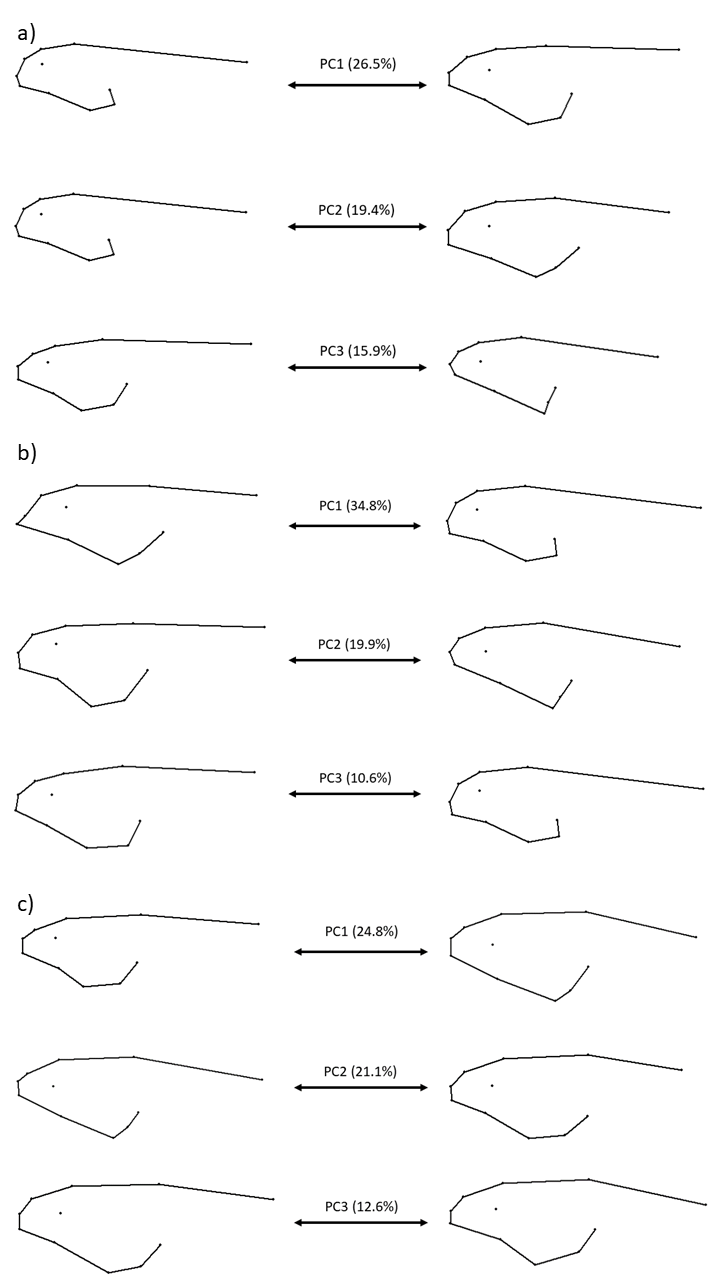


Figure S3. Craniofacial shape variation among Arctic charr (*Salvelinus alpinus*) sampled from lava caves around Lake Mývatn in 2014 and 2019. Data are presented separately for both sampling years grouped together (a), as well as 2014 and 2019 separately (b and c, respectively). Variation in craniofacial shape was assessed using 11 homologous landmarks. Superimposed landmark coordinates were subjected to a principal components analysis, where the three most informative axes were retained for each grouping. Smaller values are on the left, whereas larger values are on the right of each axis. The proportion of the total phenotypic variation explained by each axis is depicted above each arrow.
